# Histone methyltransferase WHSC1 loss dampens MHC-I antigen presentation pathway to impair IFN-**γ**–stimulated antitumor immunity

**DOI:** 10.1172/JCI153167

**Published:** 2022-04-15

**Authors:** Jiale Ren, Ni Li, Siyu Pei, Yannan Lian, Li Li, Yuchong Peng, Qiuli Liu, Jiacheng Guo, Xuege Wang, Ying Han, Guoying Zhang, Hanling Wang, Yaqi Li, Jun Jiang, Qintong Li, Minjia Tan, Junjie Peng, Guohong Hu, Yichuan Xiao, Xiong Li, Moubin Lin, Jun Qin

**Affiliations:** 1 CAS Key Laboratory of Tissue Microenvironment and Tumor, CAS Center for Excellence in Molecular Cell Science, Shanghai Institute of Nutrition and Health, Shanghai Jiao Tong University School of Medicine (SJTUSM) and Chinese Academy of Sciences, Shanghai, China.; 2 Department of General Surgery, Department of Gastroenterology, Yangpu Hospital, School of Medicine, Tongji University, Shanghai, China.; 3 Center for Clinical Precision Pharmacy, The First Affiliated Hospital, School of Clinical Pharmacy, Guangdong Pharmaceutical University, Guangzhou, China.; 4 Department of Urology, Institute of Surgery Research, Daping Hospital, Army Medical University, Chongqing, China.; 5 Department of Oncology, Shanghai Medical College, Fudan University, Shanghai, China.; 6 Department of Obstetrics, Gynecology and Pediatrics, West China Second University Hospital, Key Laboratory of Birth Defects and Related Diseases of Women and Children, Ministry of Education, Sichuan University, Chengdu, China.; 7 State Key Laboratory of Drug Research, Shanghai Institute of Materia Medica, Chinese Academy of Sciences, Shanghai, China.

**Keywords:** Oncology, Antigen presentation, Colorectal cancer, Epigenetics

## Abstract

IFN-γ–stimulated MHC class I (MHC-I) antigen presentation underlies the core of antitumor immunity. However, sustained IFN-γ signaling also enhances the programmed death ligand 1 (PD-L1) checkpoint pathway to dampen antitumor immunity. It remains unclear how these opposing effects of IFN-γ are regulated. Here, we report that loss of the histone dimethyltransferase WHSC1 impaired the antitumor effect of IFN-γ signaling by transcriptional downregulation of the MHC-I machinery without affecting PD-L1 expression in colorectal cancer (CRC) cells. *Whsc1* loss promoted tumorigenesis via a non-cell-autonomous mechanism in an *Apc^min/+^* mouse model, CRC organoids, and xenografts. Mechanistically, we found that the IFN-γ/STAT1 signaling axis stimulated WHSC1 expression and, in turn, that WHSC1 directly interacted with NLRC5 to promote MHC-I gene expression, but not that of PD-L1. Concordantly, silencing *Whsc1* diminished MHC-I levels, impaired antitumor immunity, and blunted the effect of immune checkpoint blockade. Patient cohort analysis revealed that WHSC1 expression positively correlated with enhanced MHC-I expression, tumor-infiltrating T cells, and favorable disease outcomes. Together, our findings establish a tumor-suppressive function of WHSC1 that relays IFN-γ signaling to promote antigen presentation on CRC cells and provide a rationale for boosting WHSC1 activity in immunotherapy.

## Graphical abstract

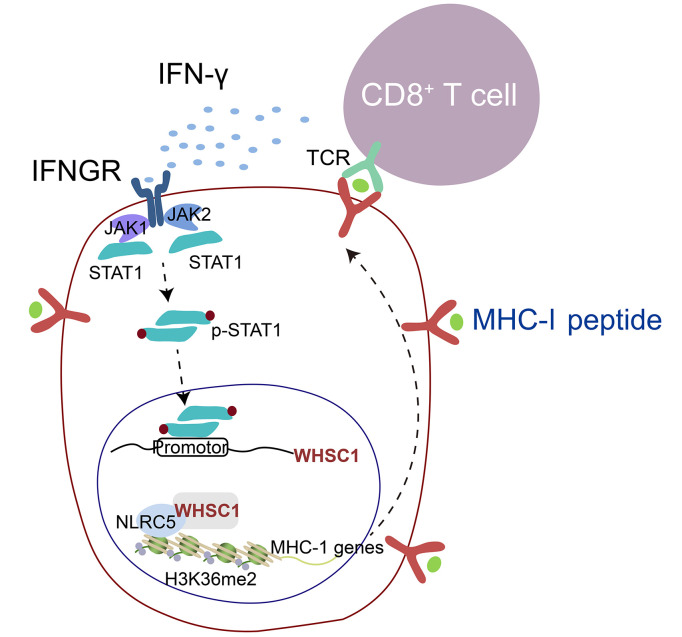

## Introduction

MHC class I (MHC-I) molecules, in complex with β-2-microglobulin (B2M), are loaded with endogenous peptides generated by the proteasome and imported into the ER by the heterodimeric TAP1/TAP2 transporter (1). A decrease in or absence of MHC-I expression results in tumor immune escape and failure of immunotherapy largely due to a lack of tumor antigen presentation to recruit and activate CD8^+^ cytotoxic T lymphocytes (2). Hence, alterations of the key components within the MHC-I antigen-processing pathway (APP) such as TAP1/TAP2 or B2M are frequently observed in colorectal cancer (CRC), melanoma, and other cancers (3–6). MHC-I downregulation occurs not only through genomic mutations but also via nongenomic mechanisms that exploit the epigenetic and transcriptional silencing of the MHC locus and/or the antigen-processing machinery (7). IFN regulatory factor 1 (IRF-1), NF-κB, and NOD-like receptor (NLR) family, caspase recruitment domain–containing 5 (NLRC5) induces MHC-I genes in response to stimulation of cytokines such as TNF-α and IFN-γ (8–10). NLRC5, also known as MHC class I transactivator (CITA), is an IFN-γ–inducible nuclear protein lacking a DNA-binding domain and is therefore tethered to the enhanceosome to occupy the MHC-I gene locus containing an SXY module (8). Reduced expression or activity of NLRC5 caused by promoter methylation, copy number loss, or somatic mutations is tightly associated with decreased MHC-I expression, impaired cytotoxic T cell activation, and unfavorable disease outcomes (11).

The interplay between active and repressive histone modifications governs gene expression and tumor development (12). In general, histone H3 lysine 27 trimethylation (H3K27me3) leads to gene repression, whereas histone H3 lysine 4 trimethylation (H3K4me3) and H3 lysine 36 methylation (H3K36me2/me3) are associated with active transcription (13). Epigenetic dysregulation is intimately associated with immune evasion. Inhibitions of DNA methyltransferases (DNMTs) and KDM1A (also known as LSD1) augment MHC-I expression via the activation of endogenous retroviruses to induce type I IFN signaling (14–16). Similarly, loss of the H3K9 methyltransferase SETDB1 de-represses transposable elements with the potential to encode viral proteins, generates MHC-I peptides, and triggers T cell responses (17). In addition, polycomb-repressive complex 2 (PRC2) silences MHC-I antigen processing, leading to the upregulation of multiple MHC-I antigen presentation genes after PRC2 inhibition (7). Thus, the downregulation of MHC-I can be compensated, at least partially, by targeting the DNA demethylation and histone methylation process, highlighting the clinical implications for targeting epigenetic machinery to enhance antitumor immunity.

Wolf-Hirschhorn syndrome candidate 1 (WHSC1) is a SET domain–containing histone methyltransferase that catalyzes the dimethylation of lysine 36 of histone H3 (H3K36me2), a mark associated with actively transcribed genes (18, 19). It has been suggested that H3K36me2 promotes transcription initiation and elongation and antagonizes polycomb silencing (20, 21). WHSC1 is associated with diseases affecting growth and development and plays a role in the DNA damage response (22, 23). In multiple myeloma, acute lymphoblastic leukemia, and prostate cancer, WHSC1 has been found to be either overexpressed or hyperactivated, resulting in increased methylation of H3K36 on promoters of oncogenes (24, 25). Consequently, WHSC1 promotes cell-cycle progression, clonogenicity, and invasion via regulation of diverse targets, depending on different genetic milieux or contexts (19, 26, 27). Nevertheless, its role as an epigenetic modifier in antitumor immunity remains largely unexplored.

The IFN-γ signaling pathway enhances MHC-I expression to stimulate antitumor immunity but also upregulates programmed death ligand 1 (PD-L1) expression (28–30). Thus, it would be ideal if the downstream pathways of IFN-γ signaling could be decoupled to selectively enhance antitumor immunity without eliciting checkpoint blockade. Here, we show that WHSC1 was induced by IFN-γ to stimulate MHC-I, but not PD-L1, expression, resulting in robust antitumor immunity.

## Results

### WHSC1 expression is negatively correlated with the disease progression of human CRC.

**Figure 1:**
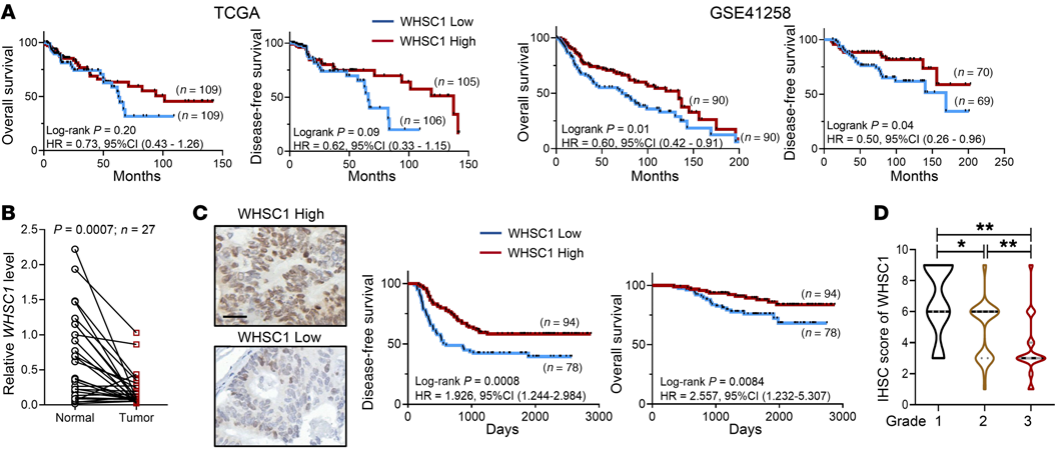
WHSC1 is negatively associated with disease outcome in CRC. (**A**) Kaplan-Meier plot of overall survival and DFS, grouped by *WHSC1* mRNA levels using TCGA and GSE41258 data sets. (**B**) Relative mRNA expression of *WHSC1* in paired normal and tumor tissues (*n =* 27). *WHSC1* expression was normalized to the mean level in the normal counterpart tissues. (**C**) Kaplan-Meier plot of overall survival and DFS stratified by WHSC1 IHC score using the Fudan TMA (*n =* 172). WHSC1 IHC scores were based on a multiplicative index of the average staining intensity (1 to 3) and the extent of staining (1 to 3). Scale bar: 50 μm. (**D**) Correlations between WHSC1 levels and tumor stages in the Fudan TMA (*n =* 172). The width of each curve in the violin plot corresponds to the approximate frequency. The solid and dotted lines show the median and quartile values, respectively, with the whiskers extending to the largest and smallest values. *P < 0.05 and **P < 0.01, by log-rank test (**A** and **C**), paired Student’s *t* test (**B**), and Kruskal-Wallis test followed by multiple comparisons (**D**).

To explore a possible role of WHSC1 in CRC, we first assessed WHSC1 expression in CRC sample from patients. We interrogated The Cancer Genome Atlas (TCGA) and GSE41258 data sets and stratified high *WHSC1* expression by median levels. We observed a trend correlating WHSC1 expression and the probability of disease-free survival (DFS) or overall survival, but it only reached statistical significance in GSE41258 but not in TCGA data sets (Figure 1A). The clinical significance of WHSC1 was supported by statistically significant downregulation of WHSC1 in colorectal tumors compared with their adjacent, normal tissue counterparts (Figure 1B). We also performed IHC staining using antibodies against WHSC1 on a tumor tissue microarray (TMA) composed of 172 patient specimens (Fudan cohort; Supplemental Table 1; supplemental material available online with this article; https://doi.org/10.1172/JCI153167DS1). Patients with lower WHSC1 levels (staining index <6) had shorter DFS and overall survival than did those with high WHSC1 expression levels (Figure 1C). In addition, WHSC1 levels were negatively associated with tumor grades in colorectal tumors (Figure 1D). Together, these findings suggest a possible role of WHSC1 in colorectal tumorigenesis.

### Whsc1 loss promotes intestinal tumorigenesis in an Apc^min/+^ mouse model.

**Figure 2:**
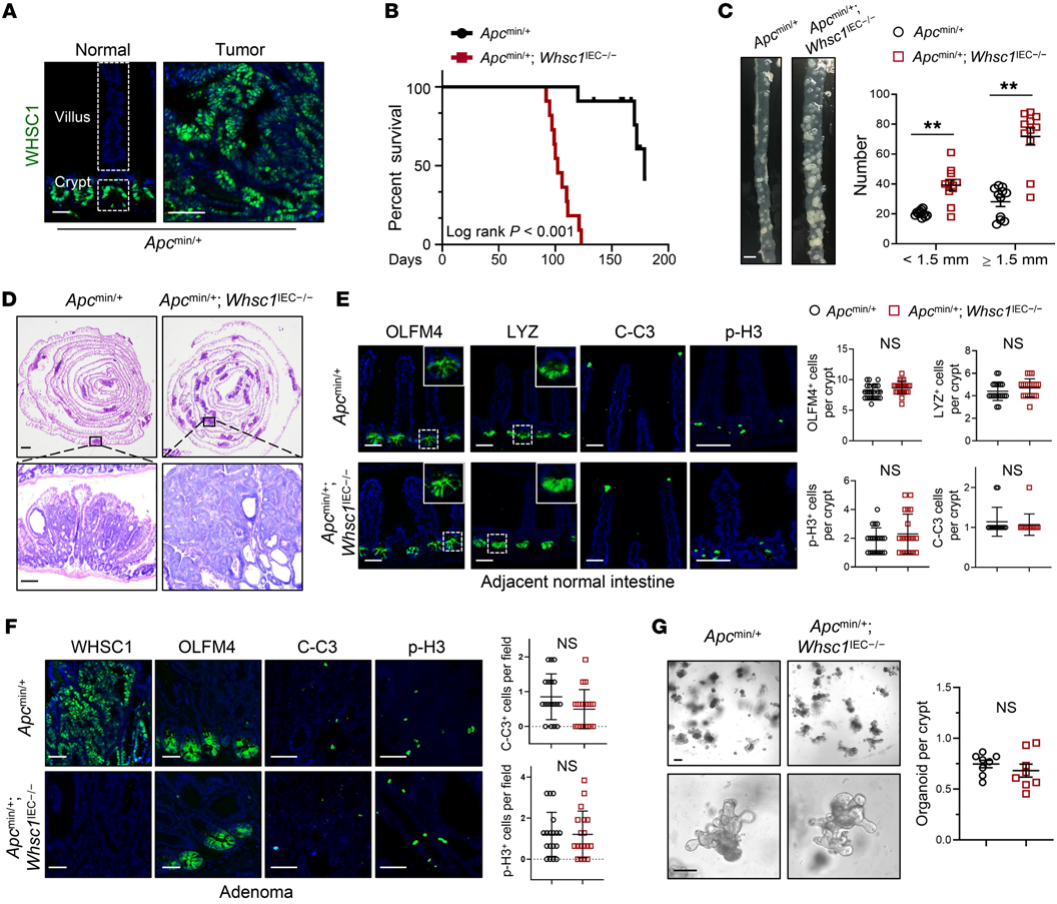
*Whsc1* deletion promotes *Apc-*mutated intestinal tumorigenesis. (**A**) Immunostaining for WHSC1 in the small intestines of 2-month-old *Apc^min/+^* mice. Scale bars: 50 μm (including insets). (**B**) Kaplan-Meier survival plots for *Apc^min/+^* and *Apc^min/+^* *Whsc1^IEC–/–^* mice (*n =* 11). (**C**) Representative images of ileum tissues from 4-month-old *Apc^min/+^* and *Apc^min/+^* *Whsc1^IEC–/–^* mice. Quantification of tumor numbers and tumor loads is shown (*n =* 11). Scale bar: 1 cm. (**D**) H&E-stained sections along the small intestine longitudinal axis in tissues from 3-month-old mice. Scale bars: 2 mm (top) and 100 μm (bottom). (**E**) Immunostaining in adjacent normal intestine from 4-month-old mice. Quantitation of the indicated cells per crypt is shown. Scale bars: 100 μm and 50 μm (enlarged insets). (**F**) Immunostaining for the indicated proteins and quantitation in adenoma from 4-month-old mice. Scale bars: 100 μm. (**G**) Representative images of organoids from *Apc^min/+^* and *Apc^min/+^* *Whsc1^IEC–/–^* mice. Quantification of the formation efficiency is shown. Scale bars: 100 μm. Data are presented as the mean ± SEM (**C** and **E**–**G**). *P < 0.05 and **P < 0.01, by log-rank test (**B**), 2-way ANOVA followed by multiple comparisons (**C**), and 2-tailed Student’s *t* test (**E**–**G**).

To explore the role of WHSC1 in intestinal tumorigenesis, we characterized WHSC1 expression in mouse intestines and found that WHSC1 protein levels were higher in the crypts and tumors of *Apc^min/+^* mice relative to those in the differentiated villus (Figure 2A). Thus, we crossed *Whsc1*-floxed mice with *Villin*-Cre mice to ablate *Whsc1* in the intestinal epithelium (*Villin^Cre/+^* *Whsc1^fl/fl^*, hereafter referred to as *Whsc1*^IEC–/–^ mice; Supplemental Figure 1A). *Whsc1^IEC–/–^* mice exhibited no gross phenotypic abnormalities over a 10-month observation period. Quantification of crypt depth and villus height of small intestine sections revealed that *Whsc1* loss did not cause appreciable alterations (Supplemental Figure 1B). The proportions of enterocytes and goblet cells by alkaline phosphatase (ALP) and periodic acid–Schiff (PAS) staining were similar (Supplemental Figure 1C). This observation was substantiated by quantitative reverse transcription PCR (qRT-PCR) analysis, showing that *Whsc1* deletion did not result in significant changes in the expression of marker genes for the differentiated cell lineages (Supplemental Figure 1D). Moreover, the levels of stem or progenitor cell–associated genes, including *Lgr5*, *Olfm4*, and *Cd44* and others, did not differ significantly between *Whsc1^fl/fl^* and *Whsc1^IEC–/–^* mice (Supplemental Figure 1D). Similar results were obtained by immunostaining for OLFM, lysozyme (LYZ), and p-H3 to examine intestinal stem cells (ISCs), Paneth cells, and proliferating cells (Supplemental Figure 1E). We found that *Whsc1^IEC–/–^* mice had no gross abnormalities in intestinal morphology under homeostatic conditions.

To determine whether ablation of *Whsc1* alters intestinal tumorigenesis, we crossed *Whsc1^IEC–/–^* mice with *Apc^min/+^* mice to generate *Apc^min/+^* *Whsc1^IEC–/–^* compound mice (Supplemental Figure 1F). Kaplan-Meier survival analysis showed that lifespan was significantly compromised in *Apc^min/+^* *Whsc1^IEC–/–^* mice. Compound mice had a median survival of 102 days, whereas *Whsc1*-intact mice had a median survival of 192 days (Figure 2B). Moreover, there was an obvious increase in the number of intestinal polyps in 3-month-old *Apc^min/+^* *Whsc1^IEC–/–^* mice compared with that seen in *Apc^min/+^* mice (Figure 2C). H&E staining further verified the increased numbers and overall sizes of the lesions in *Whsc1*-deleted mice (Figure 2D). Given that long-lived ISCs are documented as the cells of origin for intestinal tumors that develop in mice carrying *Apc* mutations (31), we assessed whether *Whsc1* loss enhances the malignant transformation of ISCs. Immunostaining analysis indicated that *Whsc1* loss did not alter the populations of ISCs or Paneth cells in the crypts of histologically normal intestines (Figure 2E). As determined by p-H3 and cleaved caspase 3 staining, the numbers of proliferative or apoptotic cells were comparable between *Apc^min/+^* and *Apc^min/+^* *Whsc*1*^IEC–/–^* mice (Figure 2E). Similar results were observed upon examination of polyps in *Apc^min/+^* and *Apc^min/+^* *Whsc1^IEC–/–^* mice (Figure 2F). These data suggest that *Whsc1* loss promoted intestinal tumorigenesis by a mechanism independent of stem cell activity and/or the growth of intestinal epithelium. An organoid model resembles the epithelial architecture of the mammalian intestine and dictates ISC activity (32). We further carried out an organoid assay and found that *Whsc1* loss did not boost the ability of the *Apc*-mutant cells to generate enteroids (Figure 2G). Likewise, whole-mount staining verified that neither proliferation nor apoptosis was altered by *Whsc1* deletion (Supplemental Figure 1G). Altogether, these findings suggest that *Whsc1* loss potentiates intestinal tumorigenesis via regulation of the tumor microenvironment.

### WHSC1 augments MHC-I expression in CRC cells.

**Figure 3:**
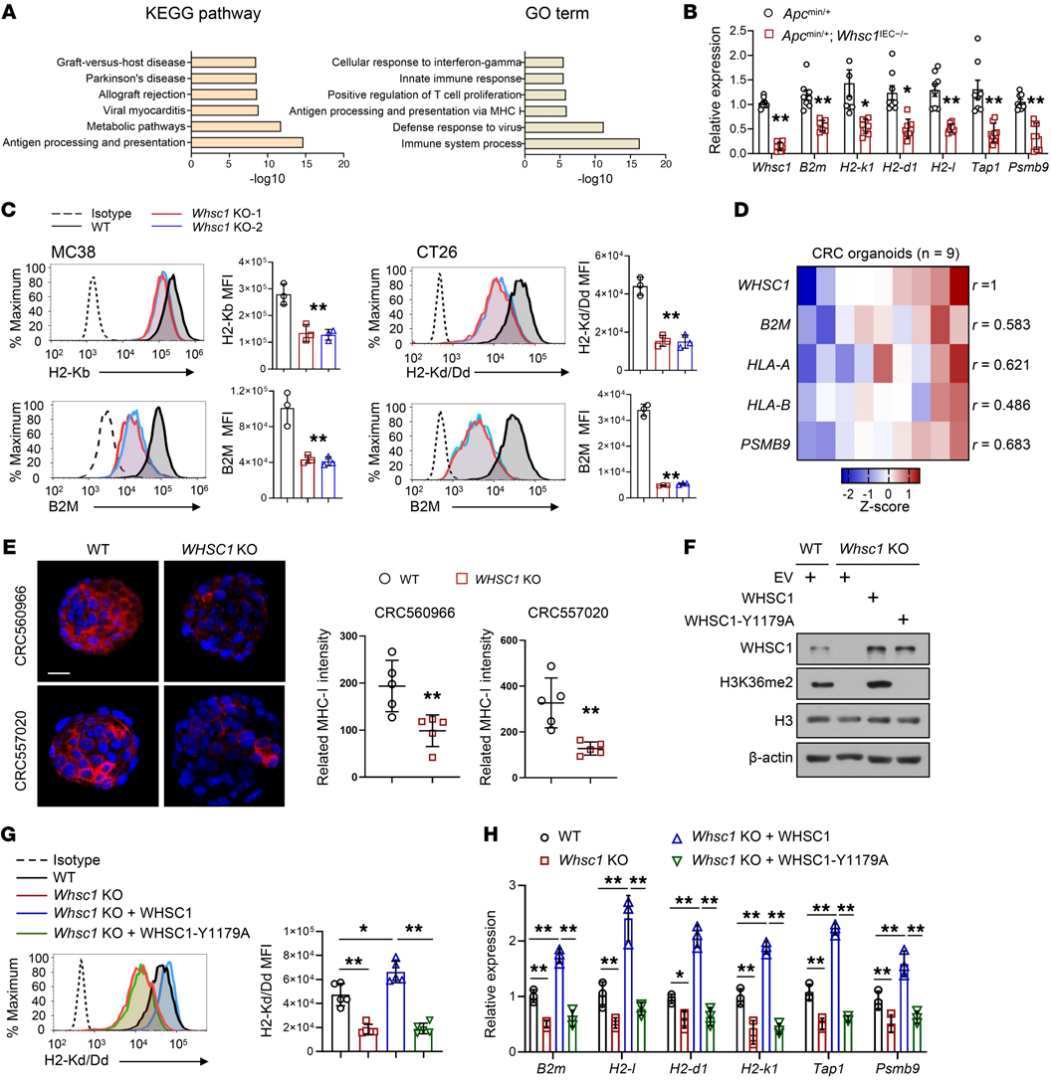
WHSC1 regulates MHC-I expression in CRC cells. (**A**) KEGG and GO analyses show the altered pathways after *Whsc1* ablation. (**B**) qRT-PCR analysis of the indicated genes in the intestinal tissues of *Apc^min/+^* and *Apc^min/+^* *Whsc1^IEC–/–^* mice (*n =* 8). (**C**) Cell-surface H2-Kb, H2-Kd, and B2M expression on MC38 and CT26 cells with or without *Whsc1* KO. The quantified MFI is shown (*n =* 3). (**D**) Heatmap summarizing the qRT-PCR results for mRNA levels of the indicated genes normalized to the mean level of each gene across all samples. (**E**) Immunostaining for MHC-I (heavy chain) in CRC organoids with or without *Whsc1* KO. MHC-I density was quantified using ImageJ (NIH). Scale bar: 20 μm. (**F**) Immunoblot (IB) analysis of *Whsc1*-KO CT26 cells with or without WT or WHSC1-Y1179A restoration. (**G**) Cell-surface levels of H2-Kd in *Whsc1*-KO CT26 cells with or without WT or WHSC1-Y1179A restoration. The quantified H2-Kd/Dd MFI is shown (*n =* 5). (**H**) qRT-PCR analysis of MHC-I–related genes in CT26 cells (*n =* 3). Data are presented as the mean ± SEM. *P < 0.05 and **P < 0.01, by 2-tailed Student’s *t* test (**B** and **E**), 1-way ANOVA followed by multiple comparisons (**C**, **G**, and **H**), and Pearson’s *R* test (**D**).

To elucidate the molecular basis by which WHSC1 inhibited intestinal tumorigenesis, we isolated morphologically normal tissues before the appearance of polyps from 6-week-old *Apc^min/+^* and *Apc^min/+^* *Whsc1^IEC–/–^* mice for transcriptomic analysis. Thus, the differences in gene expression would reflect the direct effect of *Whsc1* loss rather than secondary effects due to tumor progression. Kyoto Encyclopedia of Genes and Genomes (KEGG) pathway and Gene Ontology (GO) analyses uncovered that the most prominently altered signals or processes were associated with MHC-I antigen presentation and immunological reaction (Figure 3A). Likewise, gene set enrichment analysis (GSEA) revealed that the MHC-I signaling pathway was attenuated after *Whsc1* inhibition (Supplemental Figure 2A).

To validate these predictions, we performed qRT-PCR analysis of mouse intestines and CT26 cells and verified that *Whsc1* loss silenced the critical genes involved in MHC-I regulation, including those encoding immunoproteasome components (*Psmb9*), peptide transporters associated with antigen processing (*Tap1*), and MHC-I heavy or light chains (*H2-k1*, *H2-d1, H2-l*, and *B2m*; Figure 3B and Supplemental Figure 2B). Flow cytometry revealed that cell-surface expression of the MHC-I heavy chain (H2-Kd/Dd or H2-Kb) was approximately 2- to 3-fold lower in *Whsc1*-KO CT26 and MC38 cells than in *Whsc1*-intact cells, with similar expression levels observed for cell-surface B2M (Figure 3C). Next, we extended the analysis in human CRC cells and consistently observed that WHSC1 positively regulated MHC-I gene transcription as well as cell-surface levels of MHC-I in HCT116 and DLD-1 cells (Supplemental Figure 2, C and D). To substantiate the significance of our finding in CRC, we used patient CRC–derived organoids (patient information is enclosed in Supplemental Table 2) and identified a positive correlation between *WHSC1* levels and the expression of *B2M*, *HLA-A*, *HLA-B*, and *PSMB9* (Figure 3D). We chose organoids derived from 2 patients with the highest WHSC1 levels for subsequent assessment (CRC560966 and CRC557020; Supplemental Figure 2E). Likewise, *Whsc1 l*oss did not impair the ability of CRC cells to generate adenomatous organoids (Supplemental Figure 2F). However, immunostaining and qRT-PCR assessments of MHC-I–related molecules pointed to WHSC1 as a key regulator in the induction of MHC-I expression (Figure 3E and Supplemental Figure 2G). We showed that reintroduction of methylation-incompetent WHSC1-Y1179A–mutant in *Whsc1*-KO cells failed to restore MHC-I expression levels, indicating that the methyltransferase activity of WHSC1 is required to stimulate MHC-I expression (Figure 3, F, G, and H).

### WHSC1 loss impairs antitumor immunity via the downregulation of MHC-I expression.

**Figure 4:**
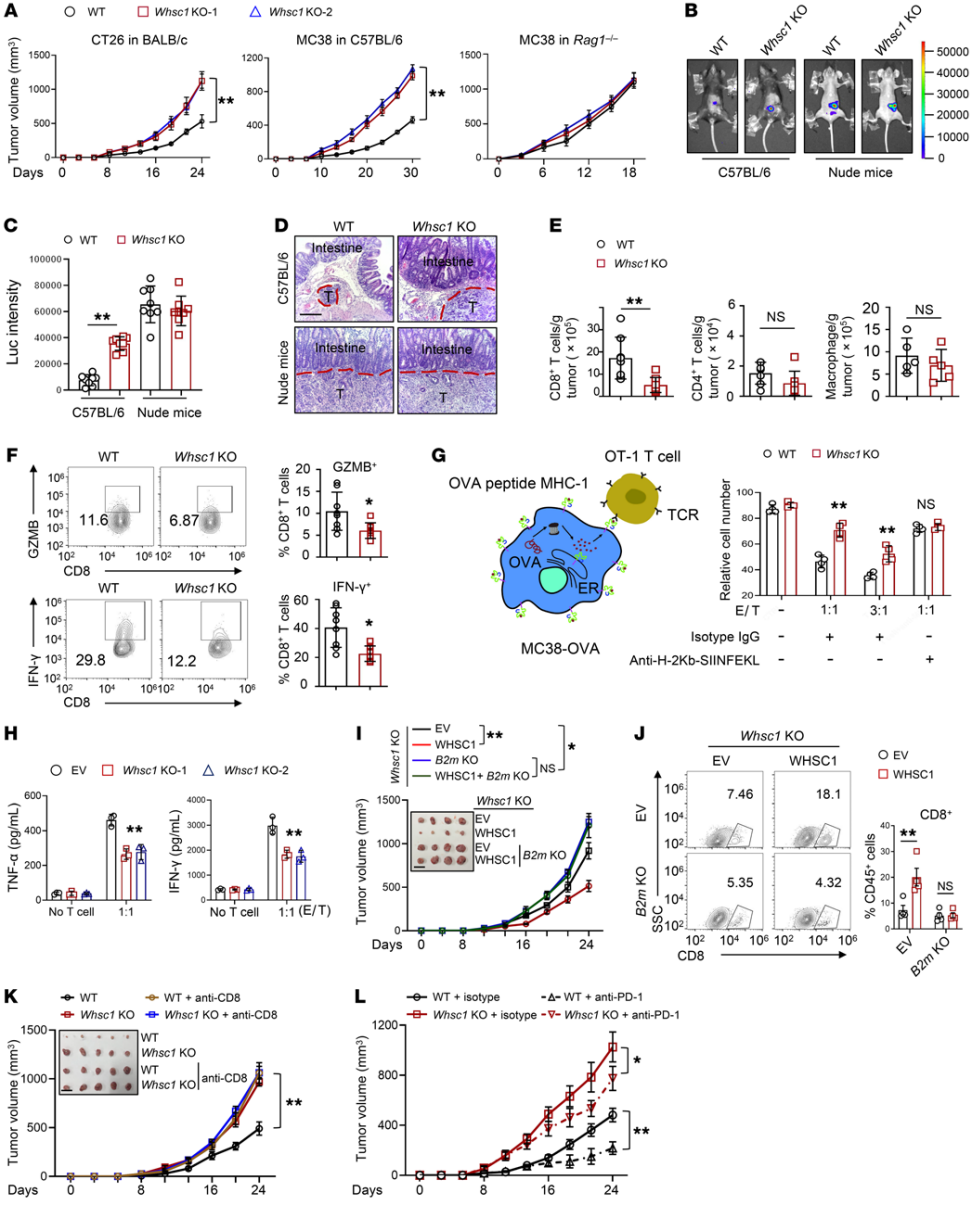
*Whsc1* KO induces resistance to antitumor immunity via MHC-I expression. (**A**) Effects of *Whsc1* loss on CT26 cell–derived (BALB/c) and MC38 cell–derived (C57BL/6 or *Rag1^–/–^*) tumor growth (*n =* 6). (**B**) Representative luminescence images of cecum xenografts derived from KAP organoids in C57BL/6 or Nude mice after 30 days of injections. (**C**) Luminescence quantification of cecum xenografts (*n =* 8). Luc, luciferase. (**D**) H&E staining of the cecum sections. Scale bars: 200 μm. (**E**) Flow cytometric analysis of CD8^+^ T cells, CD4^+^ T cells, and macrophages in tumors (*n =* 5–8). (**F**) Flow cytometric analysis of GZMB^+^ and IFN-γ^+^CD8^+^ T cells (*n =* 8). (**G**) Coculturing of OT-1 cells with MC38-OVA. After 48 hours, the viability of MC38-OVA cells was analyzed by flow cytometry (*n* = 4). Effector to target (E/T) ratios are shown. (**H**) ELISA assay for T cell effector cytokines following 48 hours of coculturing with MC38-OVA cells (*n =* 3). (**I**) Tumor growth in BALB/c mice subcutaneously injected with CT26 cells (*n =* 6). Scale bar: 1.5 cm. (**J**) Percentages of CD8^+^ T cells in tumors (*n =* 4). The numbers within the quadrants of the plot indicate the percentage of CD8^+^ T cells among total CD45^+^ cells. (**K**) CT26 tumor growth in BALB/c mice treated with anti-CD8 antibody or isotype. The mice were treated every 3 days immediately after tumor cell inoculation (*n =* 6). Scale bar: 1.5 cm. (**L**) CT26 subcutaneous tumor growth in BALB/c mice treated with anti–PD-1 antibody or isotype control antibody (*n =* 6). The mice were treated every 3 days after 8 days of tumor cell implantations. Data are presented as the mean ± SEM. *P < 0.05 and **P < 0.01, by 2-way ANOVA followed by multiple comparisons (**A** and **H**–**L**) and 2-tailed Student’s *t* test (**C** and **E**–**G**) .

We reasoned that reduced MHC-I expression might enable *Whsc1*-depleted cells to escape from CD8^+^ T cell–mediated antitumor immunity. WT and *Whsc1*-KO MC38 or CT-26 cells were subcutaneously transplanted into immunocompetent C57BL/6 and BALB/c mice, respectively. We found that *Whsc1* ablation greatly expedited tumor growth, whereas KO of *Whsc1* had minimal effects when tumor cells were engrafted into *Rag1-*null immunocompromised mice (Figure 4A), suggesting that T cells are required for WHSC1 functions. To further explore whether MHC-I downregulation evokes resistance to antitumor immunity, we established luciferase-labeled organoids derived from *Villin^Cre/+^* *Kras^G12D^* *Apc^min/+^* *Trp53^fl/fl^* (KAP) mice, which faithfully resembled the genetic alterations and disease progression in human CRC. Similarly, *Whsc1* loss significantly attenuated MHC-I expression, but did not alter the ability to generate adenoma organoids (Supplemental Figure 3, A and B). We inoculated KAP organoids into the cecal termini of C57BL/6 mice, and after 6 weeks, the mice engrafted with *Whsc1*-KO organoids produced markedly larger tumors at full penetrance. In contrast, approximately 60% (6 of 10) of the mice implanted with *Whsc1*-intact organoids developed tumors but had less malignancy (Figure 4, B–D). In a parallel assay, we did not detect a tumor-suppressive role of WHSC1 when KAP organoids were engrafted into nude mice (Figure 4, B–D).

To evaluate the tumor microenvironment, we performed flow cytometric analysis in CT26 xenografts and found decreased infiltration of CD8^+^ T cells but not CD4^+^ T cells or macrophages in *Whsc1*-deleted CT26 tumors (Figure 4E). Immunohistochemical analysis verified a profound reduction in the number of tumor-infiltrating CD8^+^ T cells, but not CD4^+^ T cells, in *Whsc1*-depleted CT26 xenografts and in the polyps of *Apc^min/+^* *Whsc1^IEC–/–^* mice compared with that seen in *Whsc1*-intact lesions (Supplemental Figure 3, C and D). Moreover, the proportion of IFN-γ^+^ or granzyme B^+^CD8^+^ (GZMB^+^CD8^+^) T cells was largely reduced in *Whsc1*-depleted CT26 xenografts or KAP-derived allografts (Figure 4F and Supplemental Figure 3E). Next, we assessed whether WHSC1-altered MHC-I expression affects tumor-associated antigen–specific (TAA-specific) CD8^+^ T cell–mediated tumor killing. In MC38 cells ectopically expressing chicken OVA protein as an antigen, intracellular processing of OVA generated the SIINFEKL peptide, which was loaded onto MHC-I and specifically recognized by OVA-specific CD8^+^ (OT-I) T cells (Figure 4G). *Whsc1* loss rendered the cells less efficient at producing OVA peptide bound to MHC-I (SIINFEKL: Kb) on the cell surface (Supplemental Figure 3F). Consequently, *Whsc1* deletion impeded T cell–mediated tumor cell–killing efficiency and produced less IFN-γ and TNF-α. Treatment with MHC-I antibody erased the difference for T cells eliciting cytotoxic effects between WT and *Whsc1*-KO cells (Figure 4, G and H), supporting the idea that the reduced antigen-specific T cell killing elicited by *Whsc1* loss was due to decreased MHC-I expression.

Moreover, we ablated *B2m* in WHSC1-overexpressing cells to determine whether the tumor-suppressive effects of WHSC1 were dependent on MHC-I upregulation. Judging by tumor volume and the frequency of CD8^+^ T cells, forced expression of WHSC1 in *Whsc1*-KO CT26 cells impeded graft growth and replenished CD8^+^ T cells compared with what we observed in the *Whsc1*-KO tumors (Figure 4, I and J). Importantly, *B2m* ablation led to the acceleration of tumor growth and the eradication of CD8^+^ T cells, irrespective of WHSC1 overexpression (Figure 4, I and J). We also depleted CD8^+^ T cells with anti–mouse CD8 mAbs in mice bearing WT or *Whsc1*-KO xenografts and found that neutralization of CD8^+^ T cells minimized the differences in tumor growth between mice bearing WT tumors and those with *Whsc*1-KO tumors (Figure 4K). We further explored how WHSC1 affected tumor immunotherapy. Compared with WT tumors, *Whsc1*-deficient tumors showed a reduced sensitivity to anti–PD-1 therapy (Figure 4L). Anti–PD-1 mAb treatment enhanced the proportion of cytotoxic CD8^+^ T cells in mice bearing WT tumors, whereas limited infiltration of GZMB^+^CD8^+^ T cell was detected in *Whsc1-*deleted tumors (Supplemental Figure 3G). Altogether, we conclude that WHSC1 loss impaired antitumor immunity.

### WHSC1 directly interacts with NLRC5 to selectively stimulate MHC-I expression.

**Figure 5:**
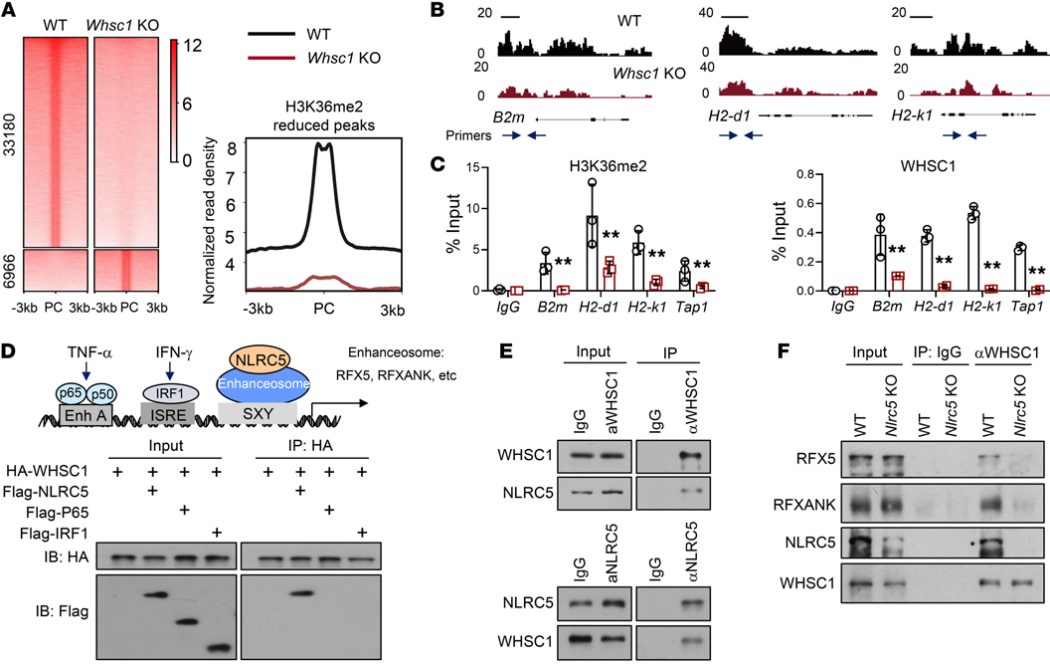
WHSC1 regulates MHC-I expression via interaction with NLRC5. (**A**) Heatmaps of H3K36me2 ChIP-Seq signals in WT and *Whsc1*-KO CT26 cells. Right panel shows quantitation of the reduced H3K36me2 signals. PC, peak center. (**B**) ChIP-Seq tracks of H3K36me2 signals at the genomic loci of *B2m*, *H2-k1*, and *H2-d1* genes. Scale bars: 1 kb. (**C**) ChIP-qPCR analysis of H3K36me2 and WHSC1 signals using the indicated primer pairs (blue arrows in **B**). (**D**) Schematic presentation of the *cis*-regulatory elements in the HLA-B promoter and IB analysis of 293T cell immunoprecipitates. (**E** and **F**) IBs analysis of CT26 cell immunoprecipitates. α, anti. Data are presented as the mean ± SEM. **P < 0.01, by 2-tailed Student’s *t* test (**C**).

Our observations that WHSC1 downregulation abrogated MHC-I expression prompted us to conduct ChIP-Seq analysis to delineate the differences in the genomic distribution of H3K36me2 modifications between WT and *Whsc1*-deficient cells. The H3K36me2 signals were preferentially enriched in intergenic and intron regions on a genome-wide scale (Supplemental Figure 4A). Comparison of H3K36me2 profiles in WT and *Whsc1*-depleted CT26 cells revealed that 5134 binding sites (2518 genes) were not significantly different, but 33,180 peaks (9061 genes) were lost and 6966 peaks (2166 genes) were gained after *Whsc1* silencing (Figure 5A). The decreased H3K36me2 modifications in MHC-I–related genes, including *B2m*, *H2-d1*, and *H2-k1* gene loci, were exemplified by browser tracts (Figure 5B). *Whsc1* deletion attenuated H3K36me2 signals together with the reduced recruitment of WHSC1 at the promoter regions of *B2m*, *H2-d1*, and *H2-k1* genes (Figure 5C). In line with the notion that H3K36me2 modification is critical for gene transcription and elongation, we observed the increase of H3K27me3 and the decrease of H3K27ac markers in the same gene loci (Supplemental Figure 4B), indicating that *Whsc1* loss results in a transcriptionally repressive chromatin for the genes associated with MHC-I expression.

We aimed to define how WHSC1 selectively induces MHC-I expression. An appealing model, we hypothesized, would be one in which the sequence-specific transcription factor recruits epigenetic regulators and guides them to its target genes. To explore this possibility, we first assessed the potential interactions between WHSC1 and key factors involved in MHC-I regulation, including IRF-1, p65, and NLRC5. Co-IP assay revealed that exogenous WHSC1 was associated with NLRC5 but not IRF-1 or p65 (Figure 5D). We further demonstrated by reciprocal co-IP that endogenous WHSC1 interacted with NLRC5 in CT26 cells (Figure 5E). Domain mapping experiments revealed that the NACHT domain within the N-terminus of NLRC5 (amino acids 210–598) was responsible for the interaction with WHSC1 (Supplemental Figure 4C). Conversely, the N-terminal fragment of WHSC1 containing the PWWP and HMG domains (amino acids 1–521) mediated the association with NLRC5 (Supplemental Figure 4D). Importantly, an in vitro binding assay demonstrated that the interaction was direct (Supplemental Figure 4E). In order to specifically induce MHC-I expression, the regulatory factor X (RFX) complex is essential for enhanceosome assembly and NLRC5 recruitment. Thus, we asked whether NLRC5 guided WHSC1 to tether with the RFX complex in the promoter regions of MHC-I genes. In line with this hypothesis, silencing of *Nlrc5* in CT26 cells attenuated WHSC1 to tether with the RFX complex, as reflected by a reduced binding affinity between WHSC1 and RFX5 or RFXANK subunits (Figure 5F).

**Figure 6:**
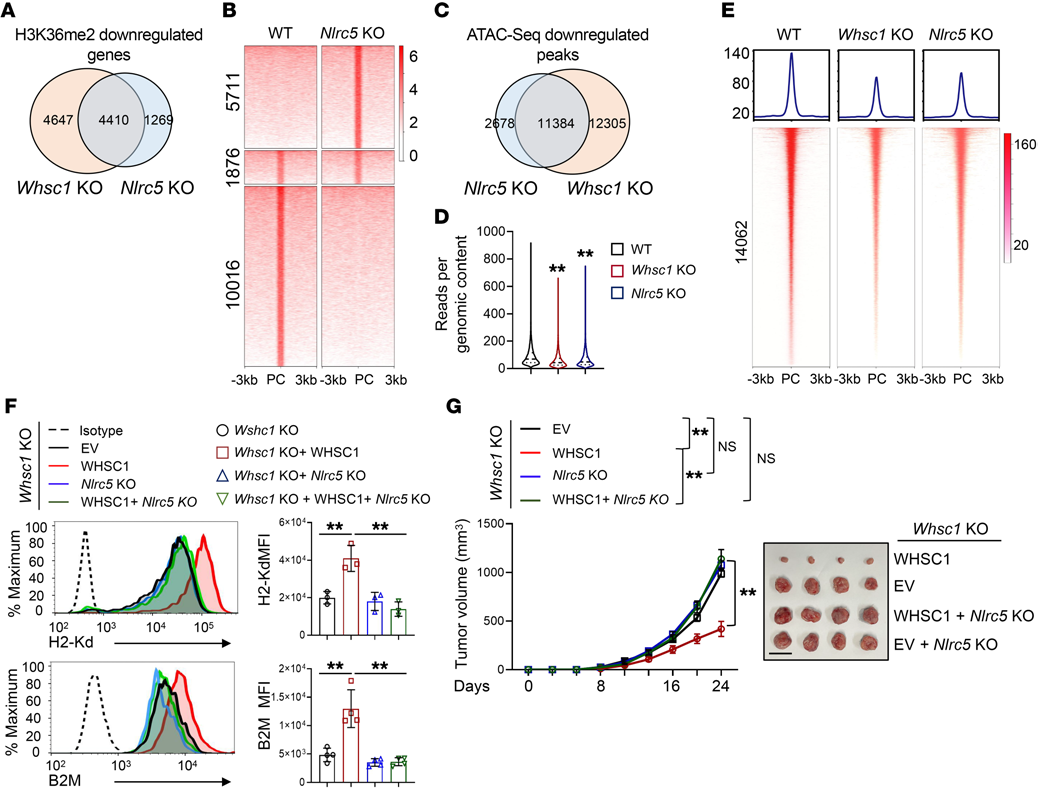
NLRC5 guides WHSC1 to the MHC-I–related gene locus but not STAT1 targets. (**A**) Venn diagram of the genes that showed reduced H3K36me2 modifications in *Whsc1*-KO and *Nlrc5*-KO CT26 cells. (**B**) Heatmaps of H3K36me2 ChIP-Seq signals for the overlapping genes in WT and *Nlrc5*-KO cells. (**C**) Venn diagram of the peaks showing reduced chromatin accessibility in *Nlrc5*-KO and *Whsc1*-KO cells compared with WT cells. (**D**) Violin plot showing ATAC-Seq signals across the peaks that lost chromatin accessibility following *Nlrc5* KO. The solid and dotted lines show the median and quartiles, respectively, with the whiskers extending to the largest and smallest values. (**E**) Heatmaps summarizing ATAC-Seq signals reduced after *Nlrc5* KO in *Whsc1*-KO, *Nlrc5*-KO, and parent CT26 cells. (**F**) Cell-surface H2-Kd or B2M and quantitation in CT26 cells (*n =* 3). (**G**) Tumor growth in BALB/c mice subcutaneously engrafted with CT26 cells (*n =* 6). Scale bar: 1.5 cm. Data are presented as the mean ± SEM. **P < 0.01, by 1-way ANOVA followed by multiple comparisons (**D**) and 2-way ANOVA followed by multiple comparisons (**F** and **G**). EV, empty vector.

To solidify the mechanistic relationship between WHSC1 and NLRC5, we conducted H3K36me2 ChIP-Seq in WT and *Nlrc5*-KO CT26 cells. *Nlrc5* deletion resulted in a total of 5679 genes that exhibited reduced H3K36me2 modifications, and 4410 of these 5679 genes also showed decreased H3K36me2 modifications in the absence of *Whsc1* (Figure 6A). Among the overlapping genes, there were a total of 17,603 H3K36me2 modification peaks and approximately 60% of them (10,016 of 17,603) had reduced levels of H3K36me2 marks after *Nlrc5* KO (Figure 6B). In addition, we performed ATAC-Seq assays and compared open chromatin regions (OCRs) in *Whsc1*- and *Nlrc5*-deleted cells. *Nlrc5* KO led to a total of 14,062 sites with reduced chromatin accessibility, and approximately 80% of the sites (11,384 of 14,062) simultaneously exhibited decreased accessibility after *Whsc1* depletion (Figure 6, C–E). Collectively, these results emphasize the functional cooperation between WHSC1 and NLRC5 on a global scale.

We further showed that WHSC1 occupancies and H3K36me2 modifications on the promoter regions of NLRC5-target MHC-I genes were significantly attenuated by the deletion of *Nlrc5* (Supplemental Figure 4F). *Nlrc5* KO led to significantly decreased H3K27ac levels and increased H3K27me3 levels at the *B2m*, *H2-d1*, *Tap1*, and *H2-k*1 gene loci, a chromatin status similar to that observed in *Whsc1*-KO cells (Supplemental Figure 4G). Next, we assessed whether WHSC1-activated MHC-I expression depends on NLRC5. Flow cytometry showed that reintroduction of WHSC1 in *Whsc1*-KO cells restored surface B2M and MHC-I levels, whereas *Nlrc5* KO abolished the effects of reintroduced WHSC1 (Figure 6F). Similar effects were observed for *B2m*, *H2-d1*, *H2-k1*, and *Tap1* (Supplemental Figure 4H). Moreover, we showed that *Nlcr5* KO abrogated the tumor-inhibitory effect induced by WHSC1 overexpression to an extent similar to that observed in *Whsc1*-KO tumors (Figure 6G). Together, NLRC5 recruited WHSC1 to stimulate MHC-I expression.

### The IFN-γ/WHSC1 axis stimulates MHC-I, but not PD-L1, expression.

**Figure 7:**
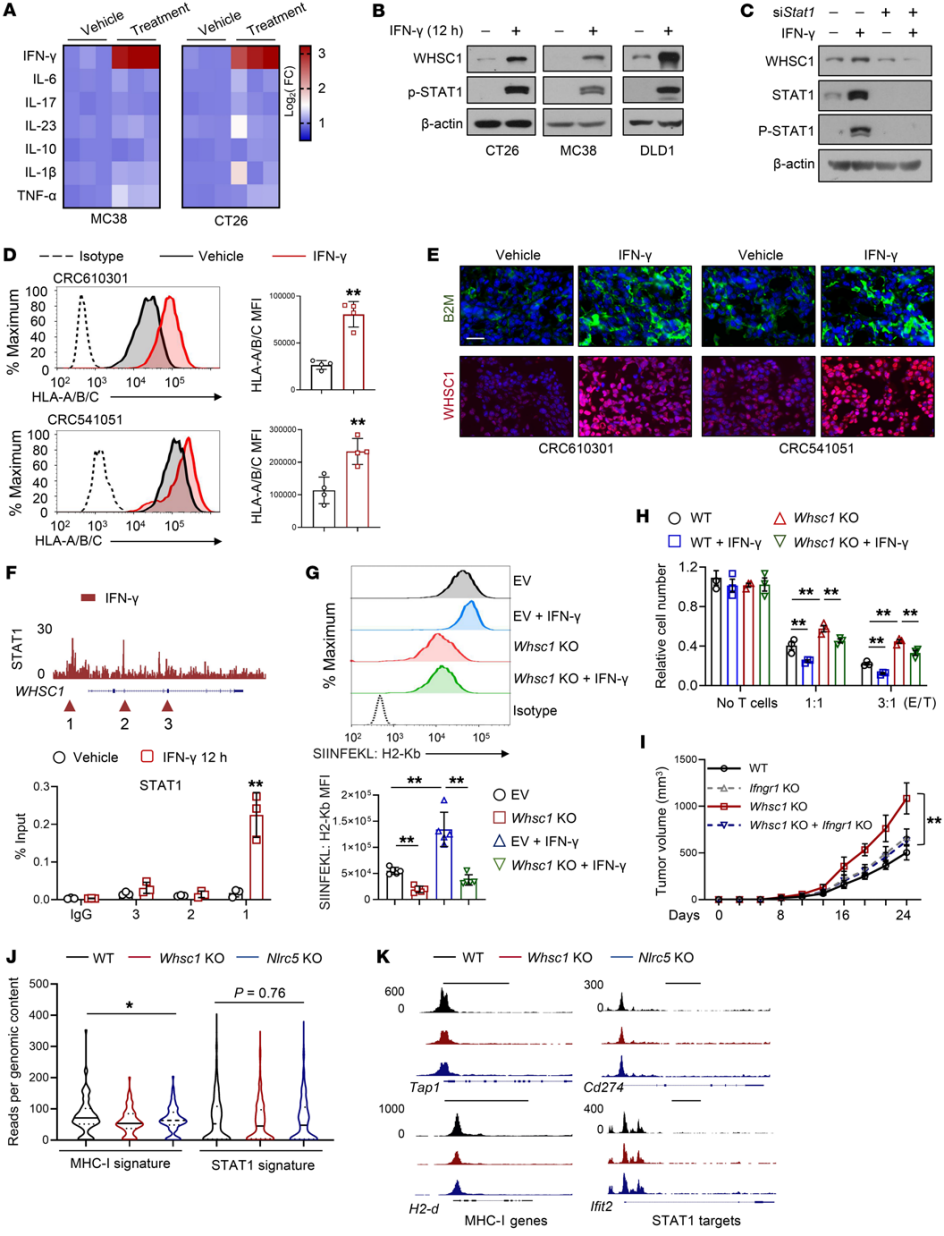
The IFN-γ/WHSC1 axis stimulates MHC-I expression. (**A**) Heatmap showing the mRNA levels of *Whsc1* in MC38 and CT26 cells treated with the indicated cytokine (24 h), normalized to the mean level of the vehicle treatment. (**B**) IB analysis of CRC cells under IFN-γ treatment. (**C**) IB analysis of CT26 cells treated as indicated. (**D**) Cell-surface HLA-A/B/C expression in the indicated CRC organoid–derived tumor lysates with or without 3 consecutive days of IFN-γ treatment (25 μg/kg, *n =* 4). (**E**) Immunostaining for B2M and WHSC1 in CRC organoid–derived tumors. Scale bar: 20 μm. (**F**) ChIP-Seq tracks of STAT1 ChIP-Seq signals at the *Whsc1* gene locus (GSE31477), and ChIP-qPCR analysis of STAT1 binding (*n =* 3). (**G**) Cell-surface SIINFEKL: H2-Kb in WT or *Whsc1*-KO MC38-OVA cells with or without IFN-γ treatment. The quantified MFI is shown (*n =* 5). (**H**) Viability of MC38-OVA cells after 48 hours of coculturing with OT-1 T cells (*n =* 3). (**I**) Tumor growth in C57BL/6 mice subcutaneously injected with WT or *Ifngr1*-KO MC38 cells with or without *Whsc1* deletion (*n =* 6). (**J**) Violin plot showing the signals across peaks for MHC-I–related genes or STAT1 targets that lost chromatin accessibility following *Whsc1* or *Nlrc5* KO. The solid and dotted lines show the median and quartiles, respectively, with the whiskers extending to the largest and smallest values. (**K**) ATAC-Seq tracks at the genomic loci of MHC-I–related genes (*Tap1* and *H2-d)* and STAT1 targets (*Cd274* and *Ifit2*). Scale bars: 5 kb. Data are presented as the mean ± SEM. *P < 0.05 and **P < 0.01, by 2-tailed Student’s *t* test (**D** and **F**), 2-way ANOVA followed by multiple comparisons (**G**–**I**), and 1-way ANOVA followed by multiple comparisons (**J**).

To characterize potential signals that govern WHSC1 expression to influence MHC-I expression, we performed a screening of CT26 and MC38 cells using a panel of cytokines, including IFN-γ, IL-6, TNF-α, and IL-1β, among others. We noticed that treatment with IFN-γ, but not the other cytokines, profoundly stimulated *Whsc1* mRNA expression in CRC cells (Figure 7A). In mouse and human CRC cells, we confirmed that IFN-γ upregulated WHSC1 expression at the protein level as well (Figure 7B). Knockdown of STAT1, a transducer of IFN-γ, abolished the WHSC1 induction elicited by IFN-γ treatment (Figure 7C). Furthermore, we took patient-derived organoids with lower WHSC1 expression (CRC610301 and CRC541051, Supplemental Figure 2E) and inoculated them into NSG mice. Treatment with exogenous IFN-γ significantly enhanced MHC-I and WHSC1 expression in CRC organoid–derived tumors (Figure 7, D and E, and Supplemental Figure 5A). To characterize the molecular basis by which IFN-γ/STAT1 promoted WHSC1 expression, we referenced STAT1 ChIP-Seq data set (Gene Expression Omnibus [GEO] GSE31477) and identified the potential STAT1-binding peaks within the *Whsc1* gene locus. ChIP-qPCR analysis revealed that STAT1 was preferably recruited to the promoter region of the *Whsc1* gene locus but not to the other loci after IFN-γ stimulation (Figure 7F). We further constructed luciferase reporters driven by the *Whsc1* promoter and found that a fragment encompassing base pairs –404 to approximately –389 harboring a conserved STAT1-binding element was directly responsible for the stimulation mediated by IFN-γ (Supplemental Figure 5B). Together, these results established that the IFN-γ/STAT1 axis transcriptionally induced WHSC1 expression.

IFN-γ is known to induce MHC-I expression via IRF-1 (33). We reasoned that WHSC1 may also transduce IFN-γ signaling to stimulate MHC-I expression independently of IRF-1. We showed that the MHC-I induction stimulated by IFN-γ was markedly abolished by *Whsc1* KO (Figure 7G). Moreover, *Whsc1* KO rendered MC38-OVA cells resistant to antigen-specific T cell killing and incompetent to produce T cell cytokines in response to IFN-γ stimulation (Figure 7H and Supplemental Figure 5C). We also generated *Ifngr1*-KO cells with or without *Whsc1* deletion. In agreement with previous studies, we found that *Ifngr1* loss did not expedite tumor growth (Figure 7I), probably because it eliminated downstream counteracting arms of IFN-γ signaling. *Ifngr1* KO led to significantly decreased WHSC1 and MHC-I levels and, importantly, abolished the tumorigenic effects elicited by *Whsc1* loss (Figure 7I and Supplemental Figure 5D). Collectively, our results demonstrate that WHSC1 transduces IFN-γ signaling to augment MHC-I expression.

Intriguingly, we found that *Whsc1* KO in CT26 cells had no detectable effect on the phosphorylation of STAT1 in response to IFN-γ (Supplemental Figure 5E), suggesting that WHSC1 did not alter the strength of IFN-γ signaling. MHC-I and CD274 (encoding PD-L1) are IFN-γ target genes. We demonstrated that *Whsc1* silencing led to a decrease in the expression of MHC-I, but not PD-L1, in response to IFN-γ treatment (Supplemental Figure 5, F and G). ATAC-Seq analysis demonstrated that WHSC1 and NLRC5 render a more accessible chromatin configuration on the MHC-I locus. In contrast, they did not change the accessibility of chromatin around STAT1 target genes (Figure 7J). The genomic snapshots highlighted the fact that MHC-I–related genes (e.g., *Tap1* and *H2d*), but not STAT1 targets such as *Cd274* and *Ifit2*, have decreased accessibility in the absence of *Whsc1* or *Nlrc5* (Figure 7K). These results indicate that WHSC1 relays IFN-γ signaling to promote antigen presentation without eliciting PD-L1 expression.

### WHSC1 expression is positively correlated with MHC-I levels in human CRC.

**Figure 8:**
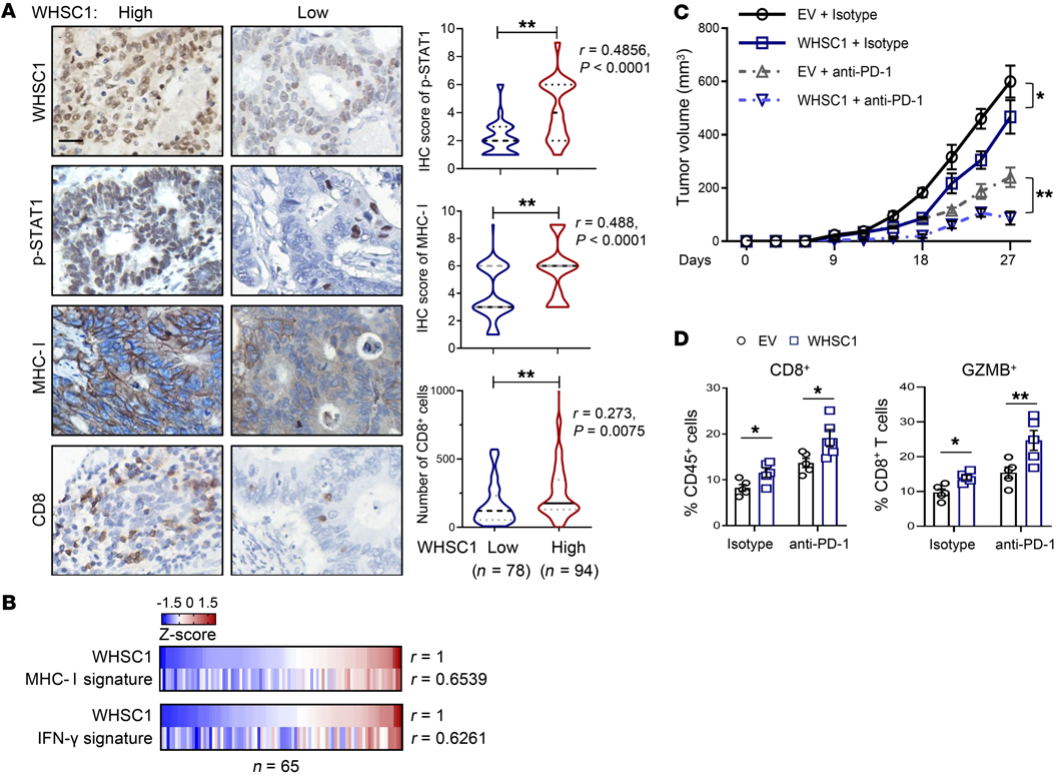
WHSC1 is positively correlated with MHC-I expression in human CRC. (**A**) The correlations between WHSC1 and p-STAT1^+^, MHC-I^+^, and CD8^+^ T cells were stratified by WHSC1, p-STAT1, and MHC-I IHC score and CD8^+^ percentage in the Fudan TMA (*n =* 172). Scale bar: 50 μm. (**B**) Heatmap summarizing the correlations (by Pearson’s test) between *WHSC1* mRNA levels and the MHC-I or IFN-γ signature (*z* score) in CRC tissues (*n =* 65). (**C**) CT26 subcutaneous tumor growth in BALB/c mice treated intravenously with anti–PD-1 or isotype control antibody every 3 days after 10 days of tumor cell implantations (*n =* 6). (**D**) Flow cytometric analysis of CD8^+^ T cells and GZMB^+^CD8^+^ T cells in tumors (*n =* 5). Data are presented as the mean ± SEM. The Wilcoxon rank-sum test was used to test the significance of immunohistochemical staining, and Spearman’s *R* test was used for correlation analysis in **A**. *P < 0.05 and **P < 0.01, by Pearson’s *R* test (**B**) and 2-way ANOVA followed by multiple comparisons (**C** and **D**).

To determine the relevance in human tumors, we performed immunohistochemical staining to measure the expression of MHC-I (heavy chain), the abundance of CD8^+^ T cells, and the activity of IFN-γ signaling reflected by phosphorylated STAT1 (p-STAT1) levels in the TMA used in Figure 1C. Patients bearing lower MHC-I levels (staining index < 6) exhibited shorter DFS and overall survival than those with high MHC-I expressions (IHC score of 6 or 9; Supplemental Figure 6A). Nevertheless, the prognostic significance of p-STAT1 level did not reach statistical significance (Supplemental Figure 6B). Importantly, we quantified that WHSC1 expression was highly correlated with p-STAT1 and MHC-I levels as well as the infiltration of CD8^+^ T cells within tumors (Figure 8A). We also analyzed the expression levels of MHC-I machinery proteins, including B2M, HLA-A, HLA-B, HLA-C, TAP1, TAP2, TAPBP, and TAPBPL, which reflect MHC-I activity, and integrated them into the MHC-I signature. Gene expression analysis showed that WHSC1 levels were positively associated with the MHC-I signature in colorectal tumors (Figure 8B and Supplemental Table 3; *n =* 65). Similarly, a strong correlation between WHSC1 and the IFN-γ–responsive gene signature (defined by expression levels of IFN-γ, STAT1, CCR5, CXCL9, CXCL10, CXCL11, IDO1, PRF1, GZMA, and HLA-DRA) was identified (Figure 8B). Together, these results emphasize the clinical significance of WHSC1 in the augmentation of MHC-I levels in CRC.

The results showing that WHSC1 selectively stimulated MHC-I expression, but not PD-L1 levels, prompted us to investigate whether increased WHSC1 expression enhances antitumor immunity. To this end, we subcutaneously transplanted WT and WHSC1-overexpressing CT-26 cells into immunocompetent BALB/c mice. WHSC1 overexpression moderately reduced tumor growth (Figure 8C). Additionally, we treated the mice with anti–PD-1 antibody when the xenografts were palpable. Compared with mice implanted with WT cells, the tumors with WHSC1 overexpression showed increased sensitivity to anti–PD-1 antibody treatment (Figure 8C). Accordingly, we detected increased infiltration of CD8^+^ T cells as well as GZMB^+^CD8^+^ T cells in WHSC1-overexpressing tumors (Figure 8D). Collectively, these results suggest that WHSC1 promotes antitumor immunity.

## Discussion

Cancer immunotherapy elicits clinical responses in some but not all patients. Efforts have largely focused on enhancing T cell functionality, but there are also alternative avenues to improve antitumor immunity by increasing tumor antigen presentation (34, 35). Our findings implicate WHSC1 downregulation as a tumor cell–intrinsic mechanism for evading antitumor immunity and resisting immune checkpoint blockade (ICB). We establish that WHSC1 promoted tumor-intrinsic immunogenicity via regulation of MHC-I expression and suggest that WHSC1 might be a reasonable biomarker to predict the clinical response to immunotherapy. Notably, T cell receptor–based (TCR-based) therapies are likely to be ineffective at eliminating immunologically cold tumors. The results showing that WHSC1 overexpression stimulated MHC-I expression and sensitized the tumor cells to anti–PD-1 mAb treatment raise the prospect that augmenting WHSC1 activity might enhance antitumor immunity. As WHSC1 is a histone methyltransferase whose enzymatic activity is potentially targetable, our results suggest that WHSC1 activation may synergize with ICB therapies, albeit agonists of WHSC1 enzyme activity have yet to be developed.

The role of WHSC1 in tumorigenesis is likely complex. Although we show that WHSC1 was required to elicit antitumor immunity, a recent study found an opposite role in prostate cancer (36). Likewise, we previously showed that WHSC1 cooperates with *Pten* deficiency to promote prostate cancer metastasis through the regulation of AKT and Rac1 signaling (25). Notably, previous studies demonstrated the cell-autonomous role of WHSC1 in promoting cell growth and invasion, albeit most results were largely based on the immunodeficient models. Here, we unexpectedly found that *Whsc1* loss did not alter cell growth in *Apc^min/+^* mice, organoids, or CRC tumors. Thus, the models used in the present study might emphasize WHSC1 function in MHC-I regulation and antitumor immunity. Taken together, WHSC1 seems to function as a molecular rheostat to balance intrinsic and extracellular clues to regulate tumor progression, depending on different genetic milieux or contexts.

Dysregulation of MHC-I and IFN-γ signaling pathways is often associated with resistance to immunotherapy (37–39). IFN-γ signaling has a dual role in antitumor immunity. It enhances MHC-I expression to stimulate antitumor immunity, but also upregulates at least PD-L1 expression to cause immune evasion (40). Although WHSC1 expression was stimulated by IFN-γ/STAT1 signaling, WHSC1 did not alter STAT1 transcriptional activity. Instead, we showed that NLRC5 recruited WHSC1 to the MHC-I–related gene locus. The specificity lies in the interaction between WHSC1 and NLRC5, in which NLRC5 modulates MHC-I genes, but not STAT1 targets. Thus, WHSC1 may be uniquely positioned as an immunogenic target for cancer immunotherapy. Notably, the present studies focused on CD8^+^ T cells, given their direct interaction with MHC-I on cancer cells. However, whether other immune cells, such as DCs, CD4^+^ T cells, and NK cells, are affected by WHSC1 awaits further investigation. For instance, as the most efficient antigen-presenting cell, DCs play essential roles in T cell priming to generate tumor-specific immune responses. We also examined whether WHSC1 modulates the MHC-I pathway in DCs to alter the priming of CD8^+^ T cells. However, our ex vivo results indicated that deletion of *Whsc1* in DCs did not affect antigen presentation to T cells (our unpublished observations), suggesting a context- or cell type–dependent role of the WHSC1/NLRC5 axis in regulating MHC-I expression. Interestingly, our preliminary bone marrow transfer results suggested that WHSC1 plays roles in immune cells to modulate antitumor immunity. Given the ubiquitous deletion of *Whsc1* in all cells within bone marrow, future studies are needed to characterize the exact types of cells in which WHSC1 exerts its functions using cell type–specific KO mouse models.

It remains poorly understood how different epigenetic regulators control the interplay between tumor cells and the immune system. Recent studies showed that PRC2 restricts the transcriptional induction of MHC-I in response to IFN-γ stimulation (7), highlighting an important aspect of the interplay between different epigenetic regulators (e.g., PRC2, WHSC1) to coordinate chromatin configurations and antigen presentation. Like most epigenetics-modifying enzymes, WHSC1 does not appear to bind to specific DNA sequences themselves. Therefore, these general chromatin-modifying enzymes are presumably recruited to specific targets in the genome by other factors to regulate specific cellular processes. At present, little is known about how WHSC1 recruitment is achieved in response to oncogenic or environmental insults. In this study, we report that NLRC5 directly bound to WHSC1 and therefore guided WHSC1 to the promoters where the CITA enhanceosome complex exists. Our results suggest a mechanism in which WHSC1 binds with a sequence-specific transcription factor by which WHSC1 is then recruited to specific sequences in the genome, thereby converting H3K36me2 to active transcription.

In summary, our results establish WHSC1 as an important cell-intrinsic regulator of antitumor immunity and suggest that pharmaceutical manipulation of WHSC1 may sensitize a subset of patients with CRC to immune checkpoint blockade.

## Methods

See the Supplemental Methods for details on the constructs, the isolation of small intestinal crypts, immunoprecipitation and immunoblotting, GST pull-down assays, RNA isolation and real-time PCR, ChIP-qPCR assays, OT-1 cell coculturing experiments, cytokine measurement, analysis of MHC-I and IFN-γ signatures, GSEA, and the primers used (sgRNAs, siRNAs, and primers; Supplemental Table 4).

### Cell culturing, infections, and transfections.

Cell lines were purchased from the American Type Culture Collection (ATCC) or Cell Bank (Shanghai Institute of Biochemistry and Cell Biology [SIBCB] of the Chinese Academy of Sciences). 293T, MC38, CT26, HCT116, and DLD1 cell lines were cultured in DMEM or RPMI-1640 with 10 % FBS at 37°C under 5% CO_2_. A lentivirus was used to establish individual stable cells, and an empty vector was used as the control for overexpression or shRNA-based knockdown. Cells were transfected with siRNA duplexes (60–100 nM) using Lipofectamine (Invitrogen, Thermo Fisher Scientific) or Dharmacon transfection reagents (MilliporeSigma) according to the manufacturers’ instructions.

### Animal experiments.

All animals were maintained in a specific pathogen–free facility. *Whsc1*-floxed mice were generated by the Beijing Biocytogen Company as previously described (25). C57BL/6-Tg (TcraTcrb) 1100Mjb/J (OT-I) mice, *Apc^min/+^* mice, and *Villin^Cre/+^* mice were purchased from The Jackson Laboratory. All mice were backcrossed with C57BL/6 mice for at least 7 generations. BALB/c, C57BL/6, *Rag1^–/–^*, or NSG male mice aged 4 to 8 weeks were injected subcutaneously with 1 × 10^6^ CT26, MC38, CRC610301, or CRC541051 cells. For cecal injections, C57BL/6 and *Rag1^–/–^* male mice aged 4 to 8 weeks were injected with 5 × 10^5^ KAP cells (derived from *Villin^Cre/+^* *Kras*^G12D^ *Apc^min/+^* *Trp53^fl/fl^* mice). An isotype or anti-CD8 antibody (10 mg/kg, Bio X Cell, BP0117) was intravenously administered every 3 days. For anti–PD-1 treatment, 10 mg/kg anti–PD-1 (Bio X Cell, BE0273) or an isotype antibody was intravenously administered every 3 days after 8 to 10 days of tumor cell implantations. For IFN-γ treatment, 25 μg/kg IFN-γ (GenScript, Z02915) was intravenously administered over 3 consecutive days after 30 days of tumor cell implantations.

### Human tumor tissues and TMA.

A TMA containing 172 CRC samples (*n* = 98 samples from male patients, *n* = 74 samples from female patients; *n* = 11 grade 1 tumors; *n* = 120 grade 2 tumors, and *n* = 35 grade 3 tumors) was constructed by the Tissue Bank of the Fudan University Shanghai Cancer Center (FUSCC). The age of patients ranged from 24 to 84 years, with a mean of 60 years and a median of 60 years. Six patients had lymph node metastasis, and 61 patients had distant metastasis. For the CRC specimens, a total of 65 samples were obtained and consisted of tumors at stage II (*n =* 29) and stage III (*n =* 36). The age of the patients (*n* = 38 males, *n* = 27 females) ranged from 33 to 90 years, with a mean of 62.2 years and a median of 63 years. The clinical parameters of the human tissue samples and TMA, including age, sex, tumor stage, pathological diagnosis, genotype, etc., are detailed in Supplemental Tables 1 and 3, respectively. The following antibodies were used: WHSC1/NSD2 (Abcam, ab75359, clone 29D1); anti–human MHC-I heavy chain (Origene, AM33035PU-N, clone HC10); and CD8 (Abcam, ab209775, clone EPR20305). Protein expression was scored and quantified by a pathologist blinded to the patients’ outcomes. The quantification method was based on a multiplicative index of the average staining intensity (1 to 3) and extent of staining (1 to 3) in the cores, yielding a 10-point staining index ranging from 1 to 9. Low expression levels of WHSC1, MHC-I, or p-STAT1 were defined by a staining index below 6, whereas staining scores if 6 to 9 were considered high expression (41).

### CRC organoids.

CRC organoids were obtained from 9 patients (tumors at stage I–II = 3, stage III = 1, and stage IV = 5). The age of the patients ranged from 46 to 81 years, with a mean of 59.4 years and a median of 58 years. The clinical information and genotype are detailed in Supplemental Table 2. CRC organoid cells were embedded in Matrigel and cultured with Advanced DMEM/F12 supplemented with 500 ng/mL Rspo1, 100 ng/mL noggin, 50 ng/mL EGF, 10 mM nicotinamide, 500 nM A830-1 (Tocris), 3 μM SB202190 (MilliporeSigma), 10 nM prostaglandin E2 (MilliporeSigma), penicillin/streptomycin, 10 mM HEPES, 2 mM GlutaMAX, 1 × B27 (Life Technologies, Thermo Fisher Scientific), 10 nM gastrin I (MilliporeSigma), and 1 mM *N*-acetylcysteine (MilliporeSigma) at 37°C under 5% CO_2_.

### IHC and immunostaining.

In brief, tissues were fixed in 4 % paraformaldehyde (PFA) and embedded in paraffin. Antigen retrieval was performed by boiling slides in citrate solution (Vector Laboratories). Slides were blocked with 5 % goat serum and incubated with a primary antibody at 4°C overnight and then with peroxidase-conjugated secondary antibodies for 1 hour. The Streptavidin-Biotin ABC Peroxidase Immunohistochemistry Kit (Vector Laboratories) was used to amplify the signal, and the antigens were stained by DAB. For immunofluorescence, after incubation with peroxidase-conjugated secondary antibodies, the slides were incubated with dye-labeled tyramide (Invitrogen, Thermo Fisher Scientific) using the following primary antibodies: anti-WHSC1 (NSD2) (Abcam, ab75359, clone 29D1); anti–human MHC-I heavy chain (Origene, AM33035PU-N, clone HC10); anti-CD8 (Abcam, ab209775, clone EPR20305); anti-lysozyme (Dako, A0099); anti–cleaved caspase 3 (Cell Signaling Technology, 9664s, clone 5A1E); anti–p-H3 (Cell Signaling Technology, 53348, clone D7N8E); anti-OLFM4 (Cell Signaling Technology, 39141, clone D6Y5A); anti–β2-microglobulin (Abcam, ab218230, clone EPR21752-214); anti-CD4 (Abcam, ab183685, clone EPR19514); and anti–p-STAT1 (Cell signaling Technology, 9167, clone 58D6). Goblet cells and enterocytes were analyzed using the Alcian Blue Periodic Acid–Schiff/AB-PAS Stain Kit (Solarbio) and the ImmPACT Vector Red Substrate Kit, Alkaline Phosphatase (Vector Laboratories), respectively.

### Lymphocyte staining and flow cytometry.

For cell-surface staining, cells were washed with staining buffer (2% FBS in PBS) and incubated with the indicated antibodies on ice for 30 minutes. For intracellular cytokine staining, cells were stimulated for 4 hours at 37°C with PMA (100 ng/mL), ionomycin (500 ng/mL), and GolgiPlug (1:1000 dilution; BD Pharmingen), followed by staining with a fixation/permeabilization buffer solution according to the manufacturer’s protocol (BD Biosciences). To determine cell viability, cells were stained with annexin V and propidium iodide (Annexin V Apoptosis Detection Kit I, BD Biosciences), and annexin V–negative cells were identified as the viable cells. The following antibodies were used: APC anti–mouse β2-microglobulin (BioLegend, 154505, clone A16041A); anti–mouse MHC-I (H-2Kd/H-2Dd) (eBioscience, 12-5998-81, clone 34-1-2S); anti–mouse MHC-I (H-2Kb) (eBioscience, 17-5958-80, clone AF6-88.5.5.3); APC anti–human HLA-A/B/C (BioLegend, 311409, clone W6/32); APC anti–mouse H-2Kb: SIINFEKL (eBioscience, 17-5743-82, clone eBio25-D1.16 [25-D1.16]); anti-CD3 (eBioscience, 17-0031-83, clone 145-2C11); anti-CD4 (eBioscience, 48-0042-82, clone RM4-5), anti-CD8 (eBioscience, 25-0081-82, clone 53-6.7); anti-CD11b (eBioscience, 47-0112-82, clone M1/70); anti-F4/80 (eBioscience, 12-4801-82, clone BM8); anti–mouse CD45, PerCP–cyanine 5.5 (eBioscience, 45-0451-82, clone 30-F11); anti–mouse IFN-γ, PE (eBioscience, 12-7311-82, clone XMG1.2); anti–mouse GZMB (eBioscience, 50-8898-82, clone NGZB); and anti–mouse Ki67, PE (eBioscience, 12-5698-80, clone SolA15).

### RNA-Seq, ChIP-Seq, ATAC-Seq, and data analysis.

Total RNA was extracted from small intestinal tissue of 6-week-old *Apc^min/+^* *Whsc1^IEC−/−^* and *Apc^min/+^* mice and then subjected to PE150 HiSeq, which was performed by the BGI Genomics Company. Each sample contained pooled RNA from 4 to 6 mice to minimize variation across samples. Transcriptome reads from the RNA-Seq experiments were mapped to the reference genome (mm10) using the Bowtie tool. Significance was set at a *P* value threshold of 0.05 and a fold change of 1.5 or greater. The differentially expressed gene were subsequently analyzed for enrichment of GO and pathways using DAVID (Database for Annotation, Visualization, and Integrated Discovery) bioinformatics and the Enrichr platform. ChIP-Seq was performed in CT26 WT and *Whsc1*-KO or *Nlrc5*-KO cells. A cross-linked pellet was prepared and followed by Magnetic ChIP (MilliporeSigma) using an antibody against H3K36me2 (Abcam). The 75 nt sequence reads generated by Illumina sequencing were mapped to the genome using the Burrows-Wheeler Aligner (BWA) algorithm with default settings. MACS2 was used to call peaks, and the default cutoff was set at a *P* value of 0.005. Density plots were generated using deepTools software (https://deeptools.readthedocs.io/en/develop/). ATAC-Seq was performed in CT26 WT and *Whsc1*-KO or *Nlrc5*-KO cells. A pellet of 50,000 viable cells was prepared, and the Vazyme TD501 Library Prep Kit (Vazyme, TD501-01) was used for tagmentation and PCR amplification. The 75 nt sequence reads generated by Illumina sequencing were mapped to the genome using the BWA algorithm with default settings. MACS2 was used to call peaks the and default cutoff was a FDR of 0.05.

### Accession codes.

RNA-Seq, ChIP-Seq, and ATAC-Seq data were deposited in the NCBI’s GEO database (GEO GSE179467, GSE179472, and GSE192672).

### Statistics.

Unless otherwise specified in the figure legends, all experiments reported in this study were performed using at least 3 mice or repeated at least 3 times independently for cells. Unless otherwise specified in the main text or figure legends, all sample numbers (*n*) represent biological replicates. Patients were classified into the low or high group, with the median expression value of all the samples used as the cutoff for Kaplan-Meier analysis. The tests used included the 2-tailed Student’s *t* test, the Kruskal-Wallis test, ANOVA, Pearson’s *R* statistical test, or the log-rank test. Data are presented as the mean ± SEM. For all statistical tests, a *P* value of less than 0.05 was considered significant.

### Study approval.

All animal experiments were performed in compliance with the *Guide for the Care and Use of Laboratory Animals* (National Academies Press, 2011) and were approved by the institutional biomedical research ethics committee of the Shanghai Institute of Nutrition and Health, Chinese Academy of Sciences. The use of clinical specimens as well as the review of all pertinent patient records were approved by the ethics committee and the IRB of FUSCC, in compliance with ethics standards and patient confidentiality. Informed consent was obtained from the patients. 

## Author contributions

JR prepared the manuscript, and JR, JQ, and NL designed the experiments. JR, NL, and SP performed most of the experiments. Q Liu, JJ, and NL performed the TMA and pathology analyses. Y Lian, LL, YP, JG, XW, YH, GZ, HW, and Y Li performed experiments and analyses. ML, XL, Q Li, MT, JP, GH, and YX supervised a specific subset of the experiments and analyses.

## Supplementary Material

Supplemental data

## Version 1 03/01/2022

In-Press Preview

## Version 2 04/15/2022

Electronic publication
