# Supplementary material for: Histone methyltransferase WHSC1 loss dampens MHC-I antigen presentation pathway to impair IFN-γ–stimulated antitumor immunity
Source: J Clin Invest. 2022 Apr 15;132(8):e153167. doi: 10.1172/JCI153167 (PMC9012282; doi:10.1172/JCI153167)
Supplement: Supplemental data [file jci-132-153167-s048.pdf]

Supplementary methods

Constructs

The full-length human WHSC1 cDNA was cloned into pLVX-puro (Flag tag) (Addgene), pMSCV (Invitrogen) to generate WHSC1 expression plasmids. The full-length human NLRC5 cDNA was cloned into pcDNA5 (HA tag) (Invitrogen) to generate NLRC5 expression plasmids. WHSC1 promoter was clone into pGL3-Basic (Promega). lentiCRISPRv2-sgRNAs targeting WHSC1, NLRC5, B2M gene locus were used to KO the genes in mouse colorectal cancer cells. The shRNA, siRNA and sgRNA sequences are listed in Supplemental Table 4.

Isolation of Small Intestinal Crypt

Intestinal organoids were derived from mouse small intestines. Briefly, the intestine was opened longitudinally, and villi were scraped away. After thorough washing, the tissues were incubated in 2 mM EDTA/PBS for 10 min and a further 15 min at 4 °C, and crypt fractions were isolated and purified by successive centrifugation steps. 100-μl of mix of Matrigel (BD Biosciences) and complete growth medium (Matrigel: complete growth medium=2:1) was added to a pellet of 50−100 crypts, and 5-μl drops of crypt-containing Matrigel were added to pre-warmed wells in a 96-well plate. After polymerization, 100 μl complete growth medium containing Advanced Dulbecco's modified Eagle's medium/F12 supplemented with 500 ng/ml Rspo1, 100 ng/ml Noggin, 50 ng/ml EGF, 10 mM HEPES, 2 mM GlutaMAX, 1× B27 (Life Technologies), penicillin/streptomycin, 1 mM N-acetylcysteine (Sigma)) was added and refreshed every 2-3 days.

Immunoprecipitation and immunoblotting

Cells were lysed with HEPES lysis buffer (20 mM HEPES, pH 7.2, 50 mM NaCl, 0.5% NP-40, 1 mM NaF and 1 mM dithiothreitol) supplemented with protease-inhibitor cocktail (Roche). Immunoprecipitations were performed using the indicated primary antibody (WHSC1/NSD2 (Abcam, ab75359), β-actin (Sigma, A3854), H3K36me2 (Abcam, ab176921), NLRC5 (Santa Cruz, sc-515668), STAT1 (Cell signalling Technology, 14994T), p-STAT1 (Tyr701) (Cell signalling Technology, 7167S), HA-HRP (Roche, 2013819), Flag-HRP (Sigma, A8592)) and

protein A/G agarose beads (Roche) at 4 °C. The immunocomplexes were then washed with HEPES lysis buffer four times. Both lysates and immunoprecipitates were examined using the indicated primary antibodies and the related secondary antibody followed by detection with the chemiluminescence substrate (Millipore).

GST pull-down assay

HA-WHSC1 protein was obtained by using in-vitro translation kit 20 (Promega). pGEX-GST-NLRC5-F2 was transformed into E. coli stain Rosetta cells. After induced by 0.5 mM isopropyl-β-D-thiogalactoside at 16 °C for 5 hours, cells were harvested in lysis buffer (50 mM Tris-HCl, pH 7.5, 150 mM NaCl, 10 % glycerol, 1 mM EDTA, 0.1 % NP-40, 1 mM DTT, 1 mM PMSF, 1 × protease-inhibitor cocktail) and sonicated (10 cycles of 15s at 30 % power). Protein was collected by GSH-coupled agarose beads and incubated with HA-WHSC1 protein. The beads were washed by 0.2 % NP-40 buffer three times and boiled by SDS-loading.

RNA isolation and real-time PCR

Total RNA was extracted using TRIzol (Invitrogen) and purified by ethanol precipitation. First strand cDNA was synthesized using the HiScript Supermix (Vazyme) and 1 μg of purified RNA for each reaction. SYBR green (Roche) and primer were used for the real-time PCR. Standard curves were generated by serial dilution of a preparation of total RNA, and all mRNA quantities were normalized against β-actin RNA. Student's t-test or One-way ANOVA (Tukey's multiple comparisons test) was used to statistical analysis of qRT-PCR results and p value less than 0.05 was considered significant. Primer sequences can be found in the Supplemental Table 4.

ChIP-qPCR assays

The ChIP assays were performed using Magnetic ChIP kit (Millipore). The procedure was as described in the kit provided by the manufacturer. Briefly, CT26 cells were fixed by 1% formaldehyde, fragmented by a combination of MNase and sonication. H3K36me2, WHSC1; H3K27ac and H3K27me3 antibody was then used for immunoprecipitation. After washing and reverse-crosslinking, the precipitated DNA was amplified by primers and quantified by the

StepOnePlus real-time-PCR machine (ABI). Primer sequences can be found in the Supplemental Table 4.

Tumor cell and OT-1 cells co-culture experiment

MC38 stably expressing OVA were dissociated into single cells, which were then incubated with either anti-H-2Kb -SIINFEKL antibody (clone 25-D1.16, BioXCell, BE0207) or isotype control (clone MOPC-21, BioXCell, BE0207) at 100 µg/ml for 30 min at 4 °C. Splenocytes were harvested from OT-I mice and stimulated by incubation with 300 ng/mL of SIINFEKL (OVA peptide) (Sigma-Aldrich) and IL-2 at 10 U/mL (Abcam) for 72 hr to expand $CD8^+$ OT-I T cells. After washing to remove the peptide, cells were cultured in media supplemented with IL-2 at 10 U/mL for an additional 2 days. $CD8^+$ T cells were enriched using MojoSor Mouse $CD8^+$ T Cell Isolation Kit (Biolegend, 480008) following manufacturer's instructions. Ten thousand MC38-OVA cells and ten or thirty thousand $CD8^+$ T cells were seeded in 96-well plates and cultured in 100 µl RPMI-1640 supplemented with 10% FBS with or without 100 µg/ml MHC-I (SIINFEKL: Kb) antibody (BioXcell, BE0207). After 48 h, the viability of MC38-OVA cells was measured by flow cytometry.

Cytokine measurement

IFN-γ and TNF-α levels were determined using a single-plex sandwich ELISA (Senxiong Biotech). The assay was performed according to the manufacturer's instructions.

MHC-1 signature and STAT1 signature

MHC_I signature is from GO_BP dataset, (http://www.gsea-msigdb.org/gsea/msigdb/geneset_page.jsp?geneSetName=GOBP_ANTIGEN_PROCESSING_AND_PRESENTATION_OF_EXOGENOUS_PEPTIDE_ANTIGEN_VIA_MHC_CLASS_I&keywords=GOBP_ANTIGEN_PROCESSING_AND_PRESENTATION_OF_EXOGENOUS_PEPTIDE_ANTIGEN_VIA_MHC_CLASS_I, gene number = 80), and STAT1 signature is form genes down-regulated in $CD8^+$ T cells: wildtype versus STAT1 knockout (https://www.gsea-msigdb.org/gsea/msigdb/cards/GSE40666_WT_VS_STAT1_KO_CD8_TCELL_DN.html, gene numb

er = 200).

Analysis of MHC-I and IFN-γ signature

MHC-I and IFN-γ signature were performed to analyze signal activity in human tumor tissues. For analysis of gene expression, we selected 10 genes directly related to MHC-I level including B2M, HLA-A, HLA-B, HLA-C, TAP1, TAP2, TAPBP, TAPBPL. Likewise, IFN-γ signature was reflected by the expression level of IFN-γ, STAT1, CCR5, CXCL9, CXCL10, CXCL11, IDO1, PRF1, GZMA and HLA-DRA. Gene expression values across all samples were converted to z-score. z-score was measured in terms of standard deviations from the mean: z-score = (value – mean)/ standard deviation. Primer sequences can be found in the Supplemental Table 4.

Gene Set Enrichment Analysis

GSEA (http://software.broadinstitute.org/gsea/index.jsp) was performed to analyze data sets involved in Reactome Class I MHC mediated antigen processing presentation from MSigDB database. Genes were pre ranked according to their expression of RNA-Seq and then ranked by GSEA software using "Differ_of_classes" and permutation type as "gene set" and other default parameters. The thresholds for inclusion were P less than 0.05 and q less than 0.25.

Figure S1

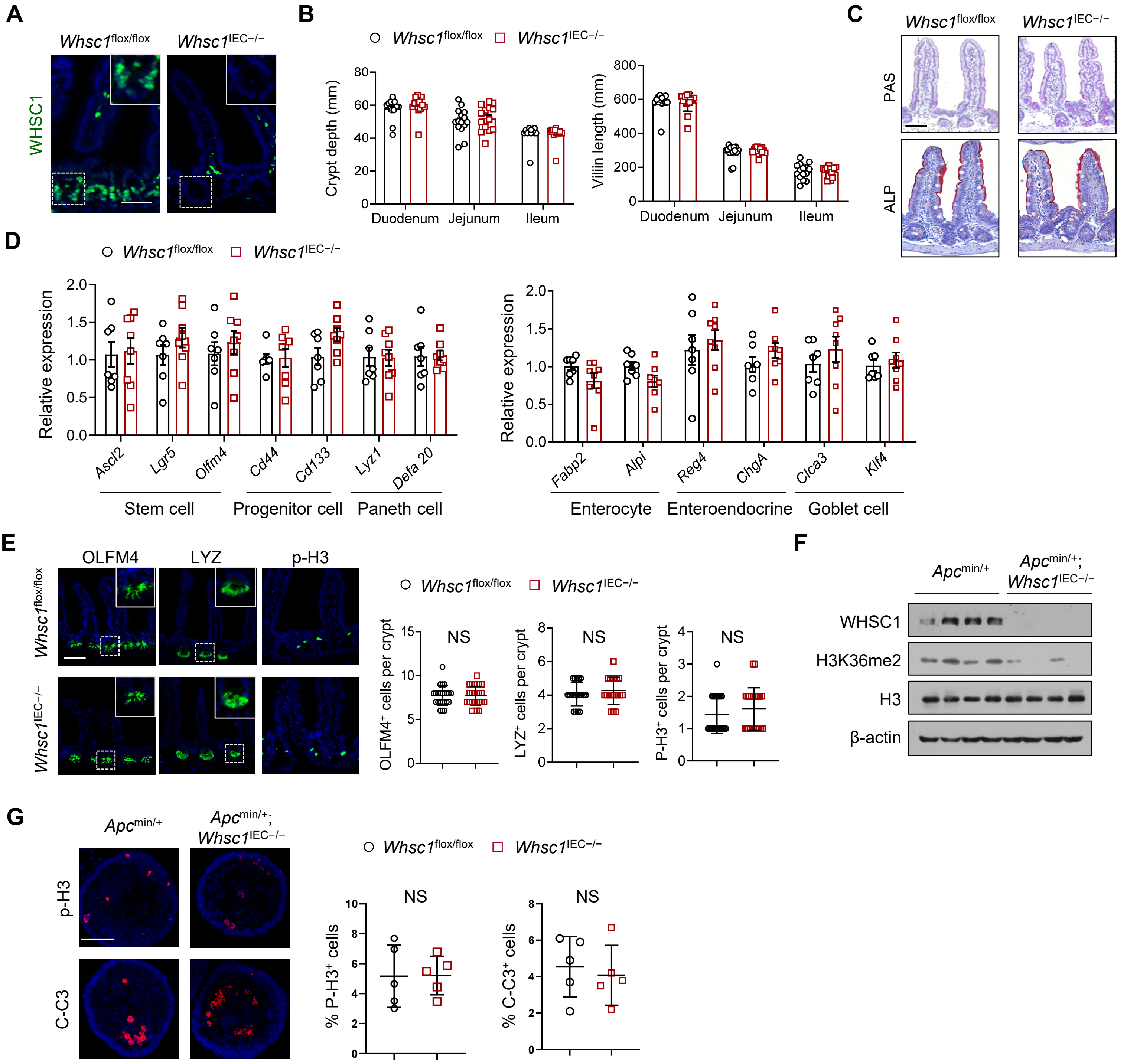

Supplemental Figure 1 *Whsc1* loss does not alter the renewal and differentiation of ISCs.

(A) Immunostaining of WHSC1 in the small intestines of 2-month-old mice. Scale bar: 50 μm.

(B) Quantification of crypt depth and villi length in duodenum, jejunum and ileum of 2-month-old mice (n = 15).

(C) ALP and PAS staining in small intestinal sections of 2-month-old mice. Scale bar: 50 μm.

(D) RT-qPCR analysis of the stem cell, progenitor cell, Paneth cell, enterocyte, enteroendocrine and Goblet cell in the small intestine of 2-month-old *Whsc1*$^{flox/flox}$ and *Whsc1*$^{IEC–/–}$ mice (n = 8).

(E) Immunostaining of indicated proteins in the small intestines of 2-month-old *Whsc1*$^{flox/flox}$ and *Whsc1*$^{IEC–/–}$ mice. The quantified OLFM4$^+$, LYZ$^+$ and p-H3$^+$ cells per crypt are shown (n = 6). Scale bar: 100 μm.

(F) IB analyses of indicated protein in small intestines from *Apc*$^{min/+}$ and *Apc*$^{min/+}$; *Whsc1*$^{IEC–/–}$ mice.

(G) Immunostaining of p-H3 and C-C3 in the organoids isolated from *Apc*$^{min/+}$ and *Apc*$^{min/+}$; *Whsc1*$^{IEC–/–}$ mice. The quantified p-H3$^+$ and C-C3$^+$ cells per crypt are shown (n = 5). Scale bar: 20 μm.

In (B), (D-E) and (G), data are presented as mean ± SEM. Statistical analyses were performed by two-tailed Student's t test (B), (D) and (G). Mann-Whitney test was used for (E). **P* < 0.05, ***P* < 0.01. NS: not significant.

Figure S2

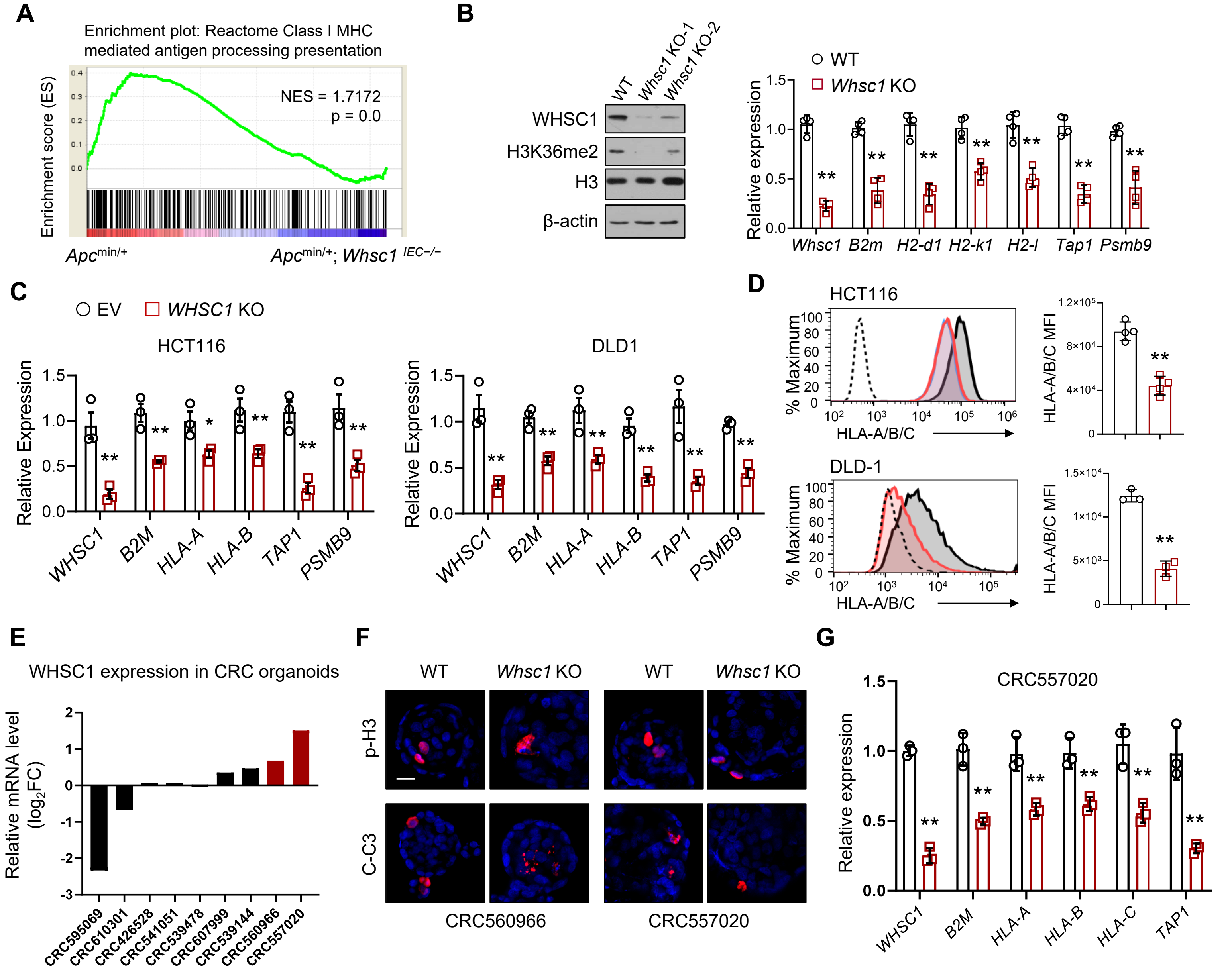

Supplemental Figure 2 WHSC1 regulates MHC-1 expression in CRC cells.

(A) GSEA enrichment plots by using public signature, Reactome Class I MHC mediated antigen processing presentation, based on all the differentially expressed genes in the absence of *Whsc1*.

(B) IB analyses of indicated protein (left) and RT-qPCR analysis of MHC-1 related genes (right) in CT26 cells (n = 3).

(C) RT-qPCR analysis of MHC-1 related genes in human colorectal cancer cell lines HCT116 and DLD1 cells (n = 3).

(D) Cell surface HLA-A/B/C in HCT116 and DLD1 cells with or without *Whsc1* KO (n = 3).

(E) mRNA level of WHSC1 in the indicated CRC organoids, normalized by mean level of WHSC1 expression across all samples. The organoids with WHSC1 high expression were shown as red columns.

(F) Immunostaining of p-H3 and C-C3 in CRC560966 and CRC557020 organoids with or without *Whsc1* KO. Scale bar: 20 μm.

(G) RT-qPCR analysis of MHC-1 related genes in CRC557020 organoids transduced with either control sgRNA or *Whsc1*-specific sgRNA (n = 3).

In (B-D) and (G), data are presented as mean ± SEM, and statistical analyses were performed by two-tailed Student's t test. *$P < 0.05$, **$P < 0.01$.

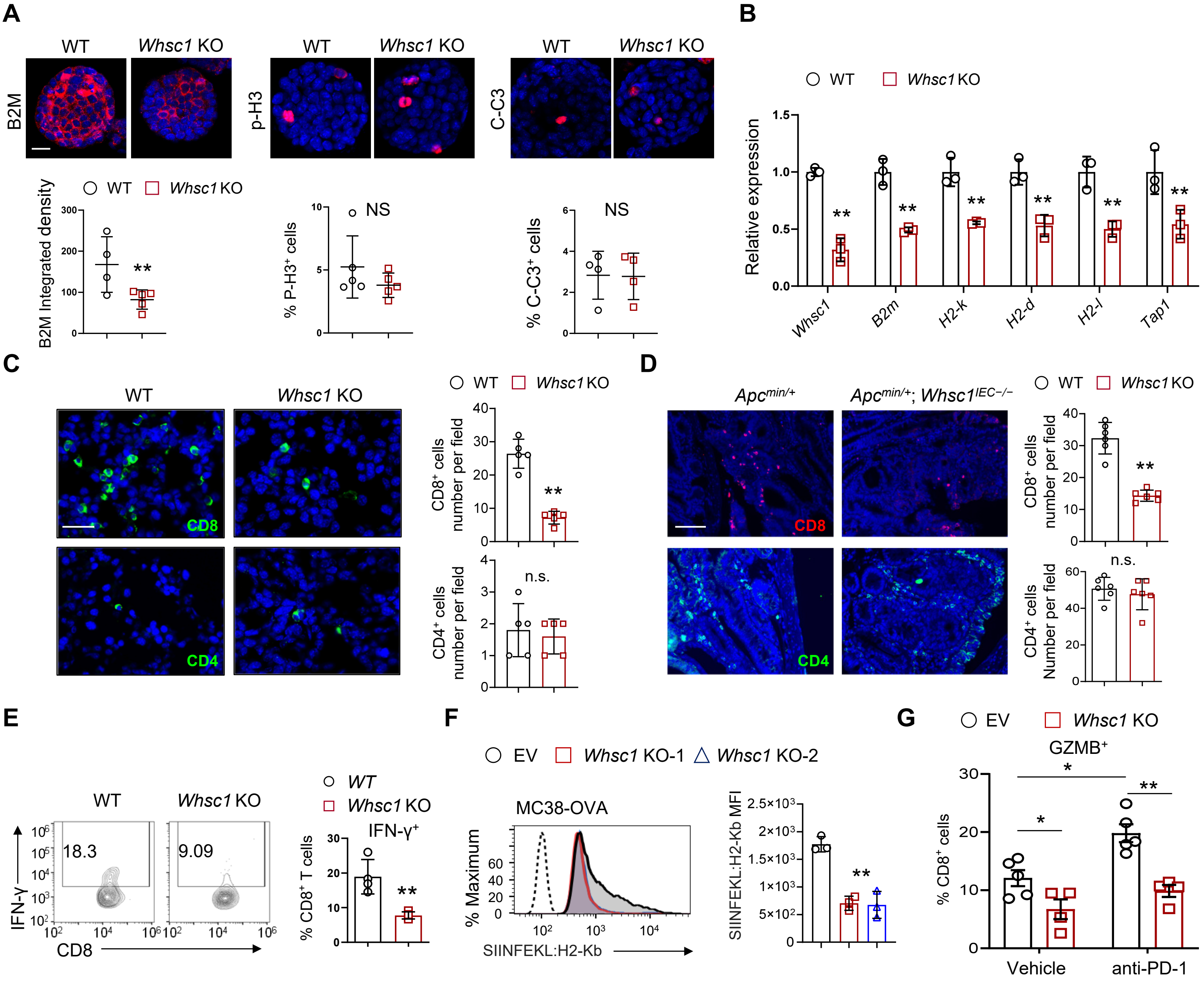

Supplemental Figure 3 WHSC1 augment MHC-1 expression to enhance anti-tumor immunity.

(A) Immunostaining of B2M, p-H3 and C-C3 in WT and *Whsc1* KO KAP organoids. The quantified p-H3^{+} cells, C-C3^{+} cells per organoid and the density of B2M are shown (n = 4). Scale bar: 20 μm.

(B) RT-qPCR analysis of MHC-1 related genes in WT and *Whsc1* KO KAP organoids.

(C-D) Immunostaining of CD8 and CD4 in WT and *Whsc1* KO CT26 tumors (C) or the polyps derived from *Apc*$^{min/+}$ and *Apc*$^{min/+}$; *Whsc1*$^{IEC-/-}$mice (D). The quantified CD8^{+} and CD4^{+} cells are shown (n = 5). Scale bar :50 μm.

(E) Flow cytometric analysis of IFN-γ^{+} CD8^{+} T cells in KAP derived tumors (n = 6).

(F) Cell surface SIINFEKL: H2-Kb in MC38-OVA cells. The quantified SIINFEKL:H2-Kb MFI is show (n = 3).

(G) Flow cytometric analysis of GZMB^{+} CD8^{+} T cells and the corresponding statistical analysis from tumors isolated from indicated mice (n = 6).

Data are presented as mean ± SEM, and statistical analyses were performed by two-tailed Student's t test for (A-E), one-way ANOVA followed by multiple comparisons for (F) and two-way ANOVA followed by multiple comparisons for (G). $*P < 0.05$, $**P < 0.01$. NS: not significant.

Figure S4

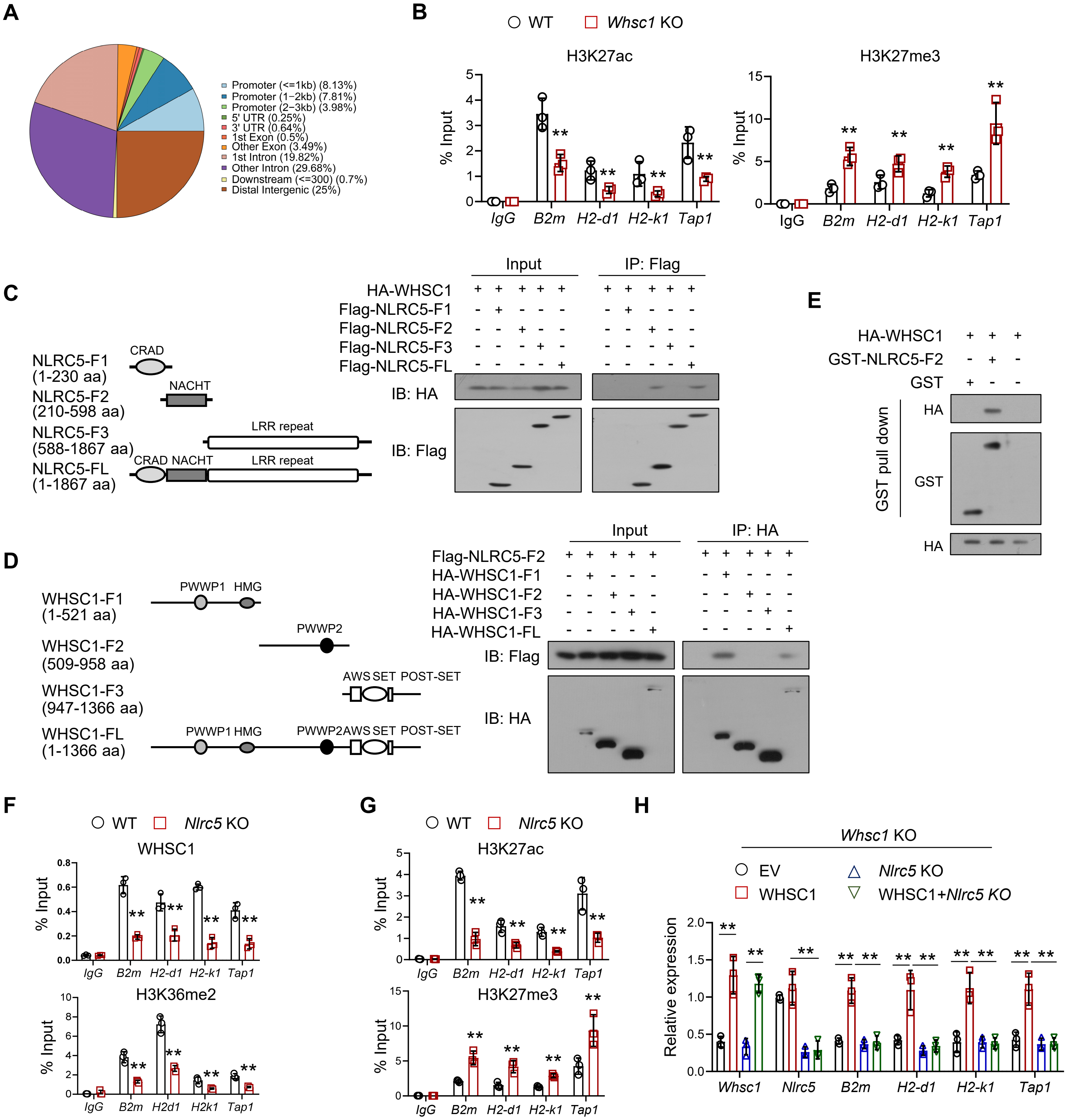

Supplemental Figure 4 NLRC5 directly interacts with WHSC1 and recruits WHSC1 to the MHC-1 gene locus.

(A) Pie chart showing the genomic distribution of H3K36me2 in CT26 cells.

(B) ChIP-qPCR analysis of H3K27ac and H3K27me3 signals as indicated (n = 3).

(C) Scheme of NLRC5 protein truncation (left) and IB analysis of immunoprecipitates of 293T cells transfected with the indicated plasmids (right).

(D) Scheme of WHSC1 truncation (left) and IB analysis of immunoprecipitates of 293T cells (right).

(E) GST pull-down analysis of the direct interaction between WHSC1 and NLRC5-F2.

(F) ChIP-qPCR of WHSC1 and H3K36me2 signals in the promoter regions of the indicated genes (n = 3).

(G) ChIP-qPCR of H3K27ac and H3K27me3 signals in the promoter regions of the indicated genes (n = 3).

(H) RT-qPCR analysis of the MHC-1 related genes in CT26 cells.

In (B and F-H), data are presented as mean ± SEM, and analyzed by two-tailed Student's t test (B, F, G) or two-way ANOVA followed by multiple comparisons for (H). $*P < 0.05$, $**P < 0.01$.

Figure S5

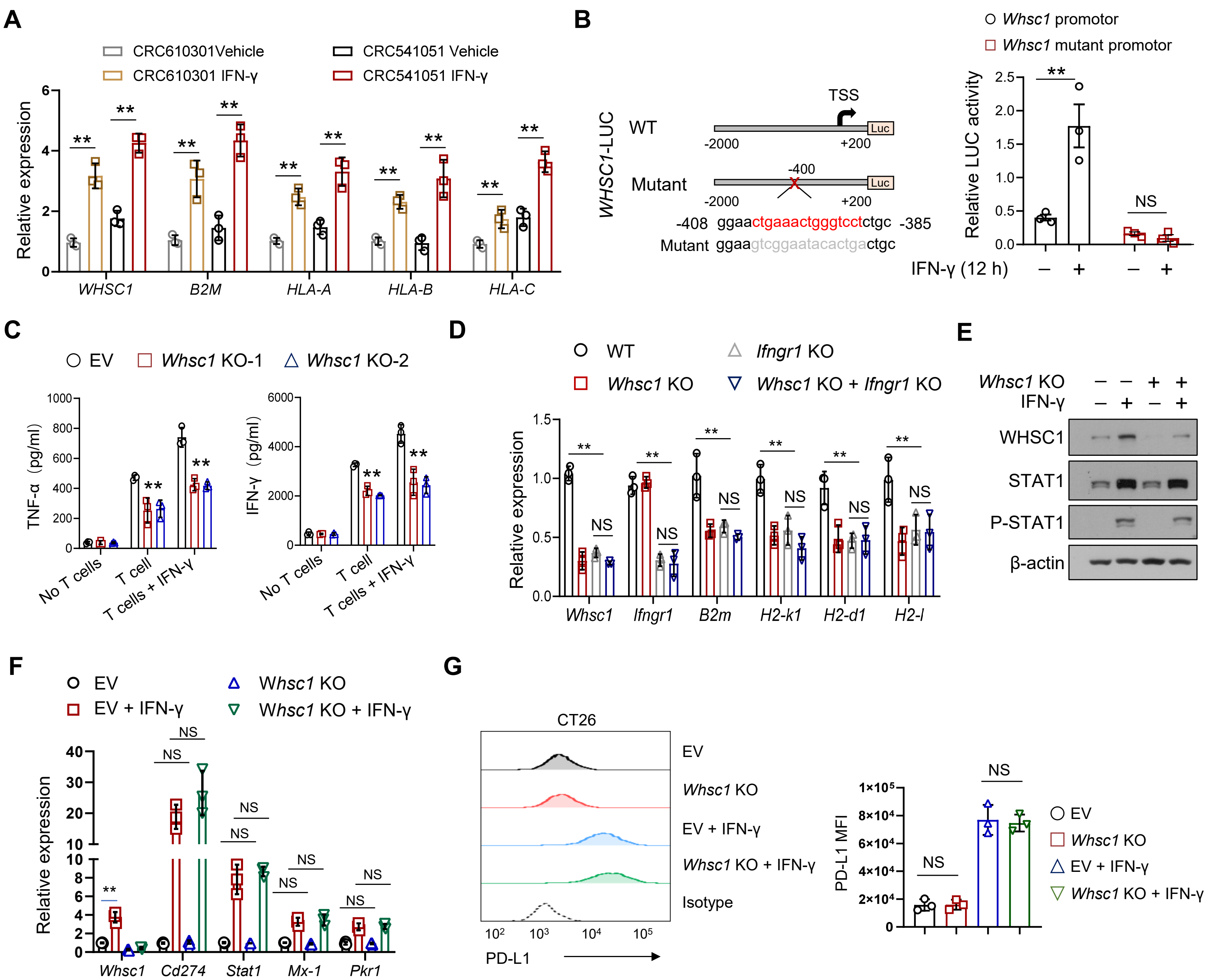

Supplemental Figure 5 *Whsc1* silencing does not affect IFN-γ signal and PD-L1 expression.

(A) RT-qPCR analysis of *WHSC*1 and MHC-1 molecules in CRC organoids derived tumors treated with IFN-γ (25 μg/kg) on 3 consecutive days (n = 3).

(B) Schematic of WHSC1 promoter-driven luciferase reporter constructs and luciferase activity in 293T cells (n = 3).

(C) ELISA assay for T cell effector cytokines following 48 h co-culture with MC38-OVA cells pretreated as indicated (n = 3).

(D) RT-qPCR analysis of MHC-1 related genes in WT or *Ifngr1* KO deleted tumors with or without *Whsc1* KO (n = 3).

(E) IB analyses of indicated protein in WT and *Whsc1* KO CT26 cells with or without IFN-γ treatment.

(F) RT-qPCR analysis of IFN-γ target genes in WT and *Whsc1* KO CT26 cells with or without IFN-γ treatment (n = 3).

(G) Cell surface PD-L1 in the indicated CT26 cells. The quantified PD-L1 MFI is shown (n = 3).

In (A-D and F-G), data are presented as mean ± SEM. Two-tailed Student's t test was used for (A), and two-way ANOVA followed by multiple comparisons for (B-D and F-G). $^*P< 0.05$, $^{**}P < 0.01$.

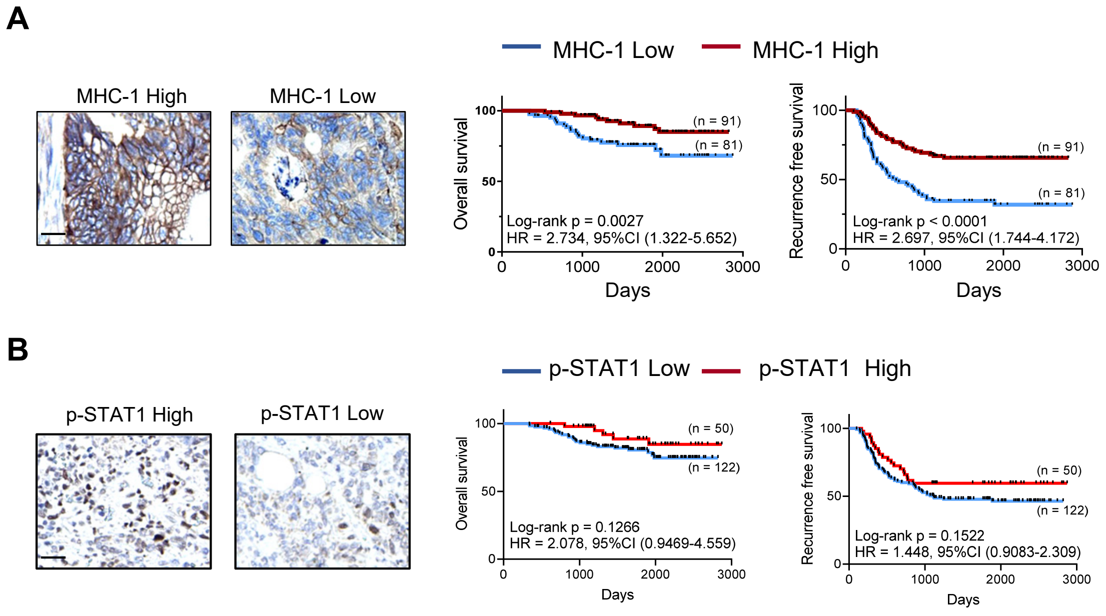

Supplemental Figure 6 WHSC1 is negatively correlated with tumor progression in human CRC.

(A) Representative images for MHC-1 (heavy chain) staining are shown in left. Kaplan-Meier plot of overall survival and DFS grouped by MHC-1 IHC score using the Fudan TMA (n = 172). Scale bar: 50 μm.

(B) Representative images for p-STAT1 are shown in left. Kaplan-Meier plot of overall survival and DFS grouped by MHC-1 IHC score using the Fudan TMA (n = 172). Scale bar: 50 μm.

Log-rank test for (A-B). *P < 0.05, **P < 0.01.

Supplemental Table 1 Clinical information of TMA

Variables	All patients
Numbers	172
Gender	
Male	98（56.97)
Female	74（43.03）
Age at diagnosis, yr	24-84（mean=60）
Location	
Colon	80（47.1）
Rectum	92（52.9）
Histology	
Adenocarcinoma	172（100）
AJCC stage	
II	172（100）
Grade	
G1-2	130（75.6）
G3	34（19.8）
Unknown	8（4.6）
Venous invation	
（-）	154（89.6）
（+）	14（8.1）
Unknown	4（2.3）
Perineural invasion	
（-）	134（77.9）
（+）	36（20.9）
Unknown	2（1.2）

Supplemental Table 2 Summary of clinical information of CRC organoids

Patient ID	Gender	Histology	Differentiation	MMR	APC	TP53	KRAS	NRAS	BRAF
CRC426528	female	Adenocarcinoma	moderately	/	/	/	G12D	/	/
CRC539144	female	Adenocarcinoma	poorly	pMMR	/	/	WT	WT	V600E
CRC539478	male	Adenocarcinoma	moderately	pMMR	/	/	WT	WT	WT
CRC541051	female	Adenocarcinoma	poorly	pMMR	/	/	G12S	WT	WT
CRC557020	male	Adenocarcinoma	moderately	pMMR	/	/	WT	WT	WT
CRC560966	female	Adenocarcinoma	poorly	pMMR	/	/	/	/	V600E
CRC595069	male	Adenocarcinoma	moderately	pMMR	/	/	WT	WT	WT
CRC607999	male	Adenocarcinoma	moderately	pMMR	/	/	WT	WT	WT
CRC610301	female	Adenocarcinoma	moderately	pMMR	/	/	WT	WT	WT

Supplemental Table 3 Clinical information of CRC Tissues

Patient ID	Gender	Age	Location	AJCC Stage	Histology	Grade	Venous invation	Perineural invasion
C130188	female	33	Sigmoid Colon	II	Adenocarcinoma	G2	(+)	(+)
C130082	male	35	Ascending Colon	III	Adenocarcinoma	G3	(-)	(-)
C130328	male	37	Rectum	II	Adenocarcinoma	G3	(-)	(-)
C130813	female	37	Sigmoid Colon	II	Adenocarcinoma	G2	(-)	(-)
C130333	male	39	Sigmoid Colon	III	Adenocarcinoma	G3	(+)	(-)
C130817	female	40	Rectum	III	Adenocarcinoma	G2	(-)	(+)
C130420	female	43	Sigmoid Colon	II	Adenocarcinoma	G2	(-)	(-)
C130482	male	43	Transverse Colon	II	Adenocarcinoma	G3	(-)	(-)
C130671	male	46	Sigmoid Colon	III	Adenocarcinoma	G2	(-)	(-)
C130692	male	48	Rectum	III	Adenocarcinoma	G2	(-)	(+)
C130234	male	48	Sigmoid Colon	II	Adenocarcinoma	G2	(-)	(+)
C130909	male	48	Ascending Colon	II	Adenocarcinoma	G1	(-)	(-)
C130006	female	49	Sigmoid Colon	III	Adenocarcinoma	G1	(-)	(-)
C130751	male	49	Ascending Colon	III	Adenocarcinoma	G3	(-)	(-)
C130561	male	50	Rectum	III	Adenocarcinoma	G2	(-)	(-)
C130952	female	51	Sigmoid Colon	II	Adenocarcinoma	G2	(-)	(-)
C130651	female	54	Sigmoid Colon	III	Adenocarcinoma	G2	(+)	(-)
C130062	female	55	Ascending Colon	III	Adenocarcinoma	G2	(-)	(-)
C130102	male	55	Ascending Colon	III	Adenocarcinoma	G3	(+)	(-)
C130259	male	55	Sigmoid Colon	III	Adenocarcinoma	G3	(+)	(-)
C130744	female	55	Sigmoid Colon	III	Adenocarcinoma	G2	(-)	(-)
C130299	female	56	Transverse Colon	III	Adenocarcinoma	G2	(+)	(-)
C130660	male	56	Rectum	III	Adenocarcinoma	G2	(-)	(-)
C130681	male	56	Sigmoid Colon	III	Adenocarcinoma	G2	(+)	(-)
C130735	male	56	Descending Colon	III	Adenocarcinoma	G2	(-)	(-)
C130986	male	56	Sigmoid Colon	II	Adenocarcinoma	G2	(-)	(-)
C130571	male	60	Sigmoid Colon	III	Adenocarcinoma	G1	(+)	(-)
C130114	male	60	Sigmoid Colon	II	Adenocarcinoma	G2	(-)	(-)
C130575	female	60	Sigmoid Colon	III	Adenocarcinoma	G2	(-)	(-)
C130841	male	60	Sigmoid Colon	II	Adenocarcinoma	G2	(+)	(-)
C130804	male	62	Ascending Colon	II	Adenocarcinoma	G1	(-)	(-)
C130527	male	63	Descending Colon	II	Adenocarcinoma	G3	(-)	(+)
C130862	male	63	Sigmoid Colon	II	Adenocarcinoma	G2	(+)	(+)
C130806	male	64	Descending Colon	II	Adenocarcinoma	G2	(-)	(-)
C130033	male	64	Sigmoid Colon	III	Adenocarcinoma	G2	(+)	(-)
C130046	female	65	Ascending Colon	III	Adenocarcinoma	G2	(-)	(-)
C130537	male	65	Ascending Colon	II	Adenocarcinoma	G2	(+)	(-)
C130264	female	66	Sigmoid Colon	III	Adenocarcinoma	G2	(-)	(-)
C130322	female	66	Sigmoid Colon	III	Adenocarcinoma	G3	(+)	(+)
C130256	male	67	Transverse Colon	III	Adenocarcinoma	G3	(+)	(+)
C130842	female	67	Transverse Colon	II	Adenocarcinoma	G2	(-)	(-)
C130101	male	68	Sigmoid Colon	III	Adenocarcinoma	G2	(-)	(-)
C130668	female	68	Descending Colon	III	Adenocarcinoma	G2	(+)	(-)
C130516	male	69	Transverse Colon	II	Adenocarcinoma	G2	(-)	(+)
C130825	male	70	Sigmoid Colon	II	Adenocarcinoma	G2	(-)	(-)
C130928	female	71	Ascending Colon	III	Adenocarcinoma	G3	(+)	(-)
C130016	female	72	Rectum	III	Adenocarcinoma	G3	(-)	(-)
C130945	male	73	Ascending Colon	II	Mucinous adenocarcinoma	G2	(-)	(-)
C130857	male	74	Ascending Colon	II	Mucinous adenocarcinoma	G3	(-)	(-)
C130452	female	74	Sigmoid Colon	III	Mucinous adenocarcinoma	G3	(+)	(-)
C130079	female	75	Ascending Colon	II	Adenocarcinoma	G2	(-)	(-)
C130129	male	76	Transverse Colon	II	Adenocarcinoma	G1	(-)	(-)
C130742	female	76	Sigmoid Colon	III	Adenocarcinoma	G2	(-)	(+)
C130381	male	77	Rectum	III	Adenocarcinoma		(-)	(-)
C130880	female	77	Transverse Colon	II	Adenocarcinoma	G2	(+)	(-)
C130518	female	79	Sigmoid Colon	III	Adenocarcinoma	G2	(-)	(+)
C130869	female	79	Sigmoid Colon	II	Adenocarcinoma		(-)	(-)
C130028	female	79	Rectum	III	Adenocarcinoma	G2	(-)	(-)
C130908	female	80	Ascending Colon	II	Mucinous adenocarcinoma	G2	(-)	(-)
C130265	male	81	Ascending Colon	II	Adenocarcinoma	G2	(-)	(-)
C130502	male	81	Ascending Colon	II	Adenocarcinoma	G2	(-)	(-)
C130069	male	81	Rectum	III	Adenocarcinoma	G2	(-)	(-)
C130745	male	84	Sigmoid Colon	III	Adenocarcinoma	G3	(+)	(-)
C130649	male	87	Descending Colon	III	Adenocarcinoma	G2	(-)	(-)
C130068	female	90	Sigmoid Colon	II	Adenocarcinoma	G3	(-)	(-)

Supplemental Table 4 Oligonucleotides

GENE	Forward	Reverse
SgMus*Whsc1*	CACCGGCACCAGCTCACATTGACAT	AAACATGTCAATGTGAGCTGGTGCC
sgMus *Nlrc5*	CACCGTAGCCTGCTAAACAACACCG	AAACCGGTGTTGTTTAGCAGGCTAC
sgMus *B2m*	CACCGATTTGGATTTCAATGTGAGG	AAACCCTCACATTGAAATCCAAATC
siMus*Whsc1*-1	CCTGGTGCTCATGATACTAAA	TTTAGTATCATGAGCACCAGG
siMus*Whsc1*-2	CACCGCGTTGTGTGGTAAATCAGTG	AAACCACTGATTTACCACACAACGC
siMus *Nlrc5*	AGAGCATCCGACTGAACAATG	CATTGTTCAGTCGGATGCTCT
siMus *Stat1*	TTGCAAGAGCTGAACTATAAC	GTTATAGTTCAGCTCTTGCAA
m*Whsc1*	GGCCAGAACAAGCTCTTACAA	TGTGGGCTCCCATAAAAGCTC
m*Ki67*	ATCATTGACCGCTCCTTTAGGT	GCTCGCCTTGATGGTTCCT
m*Ccnd1*	GCGTACCCTGACACCAATCTC	CTCCTCTTCGCACTTCTGCTC
m*Cd133*	CCTTGTGGTTCTTACGTTTGTTG	CGTTGACGACATTCTCAAGCTG
m*Ascl2*	AAGCACACCTTGACTGGTACG	AAGTGGACGTTTGCACCTTCA
m*Lgr5*	CCTACTCGAAGACTTACCCAGT	GCATTGGGGTGAATGATAGCA
m*Olfm4*	CAGCCACTTTCCAATTTCACTG	GCTGGACATACTCCTTCACCTTA
m*Cd44*	TCGATTTGAATGTAACCTGCCG	CAGTCCGGGAGATACTGTAGC
m*Reg4*	GGCGTGCGGCTACTCTTAC	GGAAGTATCCATAGCAGTGGGA
m*Chga*	ATCCTCTCTATCCTGCGACAC	GGGCTCTGGTTCTCAAACAC
m*Anpep*	ATGGAAGGAGGCGTCAAGAAA	CGGATAGGGCTTGGACTCTTT
m*Fabp2*	GTGGAAAGTAGACCGGAACGA	CCATCCTGTGTGATTGTCAGTT
m*Alpi*	ATGATGCCAACCGAAACCCC	GCGTGTCCTTCTCATTGGTAA
m*Defa rs1*	CCAGGCTGTTTCTGTCTCCT	CCTTTCTTCGCACAATGGCT
m*Defa 20*	TGTAGAAAAGGAGGCTGCAATAG	AGAACAAAAGTCGTCCTGAGC
m*Lyz1*	GAGACCGAAGCACCGACTATG	CGGTTTTGACATTGTGTTCGC
m*Clca3*	CTGTCTTCCTCTTGATCCTCCA	CGTGGTCTATGGCGATGACG
m*Klf4*	GTGCCCCGACTAACCGTTG	GTCGTTGAACTCCTCGGTCT
m*B2m*	TTCTGGTGCTTGTCTCACTGA	CAGTATGTTCGGCTTCCCATTC
m*H2-k1*	CAGGTGGAGCCCGAGTATTG	CGTACATCCGTTGGAACGTG
m*H2-d1*	TCTCTGTCGGCTATGTGGAC	TCTGCTGGAGTGTGTGAGAG
m*Tap1*	GGACTTGCCTTGTTCCGAGAG	GCTGCCACATAACTGATAGCGA
m*Stat1*	TCACAGTGGTTCGAGCTTCAG	GCAAACGAGACATCATAGGCA
m*Mx-1*	GACCATAGGGGTCTTGACCAA	AGACTTGCTCTTTCTGAAAAGCC
m*Pkr-1*	ATGCACGGAGTAGCCATTAC	TGACAATCCACCTTGTTTTCGT
m*Cd274*	GCTCCAAAGGACTTGTACGTG	TGATCTGAAGGGCAGCATTTC
h*B2M*	GAGGCTATCCAGCGTACTCCA	CGGCAGGCATACTCATCTTTT
h*HLA-A*	GACGCCCCCAAAACGCATA	TGGGCAAACCCTCATGCTG
h*HLA-C*	TCATCTCAGTGGGCTACGTG	GTCCTCGCTCTGGTTGTAGT
h*HLA-B*	CAGTTCGTGAGGTTCGACAG	CAGCCGTACATGCTCTGGA
h*TAP1*	TGCCCCGCATATTCTCCCT	CACCTGCGTTTTCGCTCTTG
h*TAP2*	TGGACGCGGCTTTACTGTG	GCAGCCCTCTTAGCTTTAGCA
h*TAPBP*	TGGACCGGAAATGGGACCT	CCCCAGAAGGGTAGAAGTGG
h*TAPBPL*	TGCCCGGCCTCACTATACA	GCTCAGTCGTACTTTAGGGGAAG
h*IFNG*	TCGGTAACTGACTTGAATGTCCA	TCGCTTCCCTGTTTTAGCTGC
h*STAT1*	CAGCTTGACTCAAAATTCCTGGA	TGAAGATTACGCTTGCTTTTCCT
h*CCR5*	GTTGGACCAAGCTATGCAGGT	GCAGAAGCGTTTGGCAATGT
h*CXCL9*	CCAGTAGTGAGAAAGGGTCGC	AGGGCTTGGGGCAAATTGTT
h*CXCL10*	GTGGCATTCAAGGAGTACCTC	TGATGGCCTTCGATTCTGGATT
h*CXCL11*	GACGCTGTCTTTGCATAGGC	GGATTTAGGCATCGTTGTCCTTT
h*IDO1*	TCTCATTTCGTGATGGAGACTGC	GTGTCCCGTTCTTGCATTTGC
h*PRF1*	GTGGGACAATAACAACCCCAT	TGGCATGATAGCGGAATTTTAGG
h*GZMA*	CCCTATCCATGCTATGACCCA	AGTATCGGACCAAGATGCACTAT
h*HLA-DRA*	ATACTCCGATCACCAATGTACCT	GACTGTCTCTGACACTCCTGT
ChIP*B2m*（-2k）	AAATCTCCACATCCAGAAAGCT	ATCGTGGGGAAACTGAGAGG
ChIP*H2-k1*（-2.7k）	AAGAGCCCACTATCAGCCTC	GAGAGCACGTGTTCCTGTTG
ChIP*H2-d1*（-0.8k）	TAGCCCCACTCCTCCTAACA	AGGAGAGCAAGCAAGGGAAT
ChIP*Tap1*（-0.2k）	GGCTCGGCTTTCCAATCAG	TGCTTGCGTGAATTTTCCCA
ChIP*Whsc1*（-0.3k）	ACAAGTGTGCCTAGTGTCTG	AAGGGAAAGTAGTCGGGACA
ChIP*Whsc1*（+15k_	CATGTATGCAGGCAAACACT	TATAGTCCTGACTATCCTGG
ChIP*Whsc1*（+30k）	CACACAGACTAGCTCAGTCA	AATGCTAGTGTAAATACGGG
